# Synthesis and Ring-Opening Metathesis Polymerization of *o*-Dialkoxy Paracyclophanedienes

**DOI:** 10.1021/acs.macromol.2c02111

**Published:** 2022-12-07

**Authors:** Yurachat Janpatompong, Andrew M. Spring, Venukrishnan Komanduri, Raja U. Khan, Michael L. Turner

**Affiliations:** Department of Chemistry, University of Manchester, Oxford Road, Manchester M13 9PL, U.K.

## Abstract

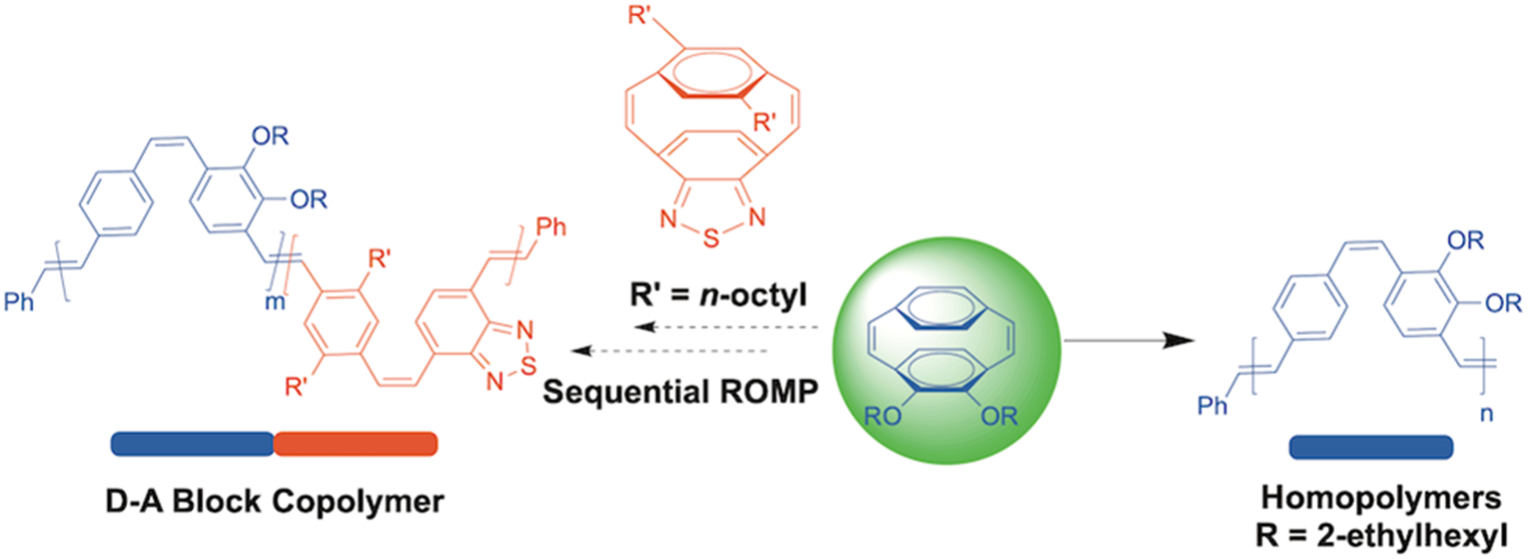

The highly strained *ortho*-diethylhexyloxy [2.2]paracyclophane-1,9-diene (**M1**) can be synthesized by ring contraction of a dithia[3.3]paracyclophane using a benzyne-induced Stevens rearrangement. This paracyclophanediene undergoes ring-opening metathesis polymerization to give well-defined 2,3-dialkoxyphenylenevinylene polymers with an alternating *cis*/*trans* alkene stereochemistry and controllable molecular weight. Fully conjugated block copolymers with electron-rich and electron-deficient phenylene vinylene polymer segments can be prepared by sequential monomer additions. These polymers can be readily isomerized to the all-*trans* stereochemistry polymer. The optical and electrochemical properties of these polymers were investigated by theory and experiment.

## Introduction

π-Conjugated organic polymers display promising optical and electronic properties^1−5^ for application in organic light-emitting diodes (OLEDs),^6,7^ organic photovoltaic cells (OPVs),^8^ organic field effect transistors,^9^ fluorescent imaging agents, or biological and chemical sensors.^10^ Phenylene vinylene polymers (PPVs) are one of the first semiconducting polymers reported, and extensive research into the synthesis and physical properties of these materials has been conducted.^11,12^ These polymers were used in the active layer of the first polymeric OLED^13^ and the repeating unit remains of interest for application in emissive devices,^14,15^ transistors,^16,17^ OPVs,^18^ and novel areas such as bioimaging^19−21^ and drug delivery systems.^22^

The unsubstituted parent polymer, poly(*p*-phenylenevinylene), is completely insoluble, intractable, and infusible, making direct solution processing of this material impossible.^23^ The incorporation of side chains on the polymer backbone can enable the solution processing of these materials. Introduction of alkoxy substituents, generally in the 2,5-positions of the phenylene ring, leads to a considerable bathochromic shift in the absorption and emission of these materials due to the donation of electron density, raising the HOMO energy of the π-conjugated backbone.^24,25^

**Scheme 1:**
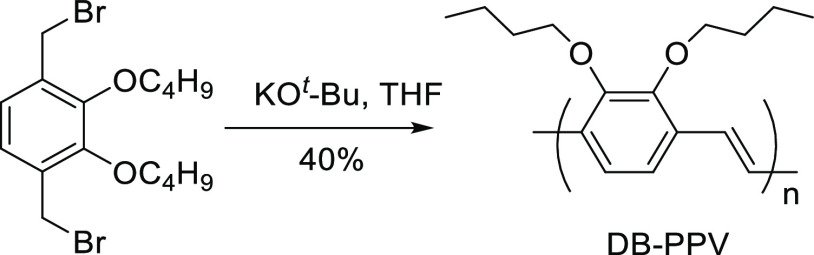
Synthesis of 2,3-Dialkoxy-1,4-PPV

PPVs can be prepared by step-growth polymerization methods (e.g., palladium-catalyzed Stille, Suzuki, and direct arylation).^26−29^ These versatile approaches lead to diverse backbone structures but provide poor control of molecular weight, broad dispersities (*Đ*), and ill-defined end groups. The simplest approach to the preparation of the most commonly reported 2,5-dialkoxy substituted PPVs is the dehydrohalogenation of 1,4-*bis* (bromomethyl)-2,5-dialkoxybenzenes.^30^ This reaction (the Gilch reaction) proceeds by chain growth radical and anionic mechanisms to give high-molecular-weight polymers but with poor control of the dispersity and the incorporation of saturated backbone defects. The Gilch methodology has also been used to prepare 2,3-dialkoxy-substituted phenylenevinylene polymers (Scheme 1); in these polymers every phenylene is functionalized with two adjacent alkoxy groups but the polymers are isolated with a broad dispersity (*Đ*) and a variety of end groups.^31^

Alternative synthetic approaches have been developed for PPVs with less defective polymer backbones, control of the molecular weight, narrow *Đ*, and well-defined end groups. One such method is the ring-opening metathesis polymerization (ROMP) of strained cyclic alkenes, such as the paracyclophane-1,9-dienes.^32^ Effective initiators are ruthenium carbene complexes, and the living nature of the polymerization enables the preparation of block copolymers, including polymers with fully conjugated donor–acceptor (D–A) blocks.^33−37^ Alternation of the donor and acceptor moieties within the polymer backbone leads to narrower band gaps for these copolymers.^38−40^ To date, 2,5-substituted polymers have been exclusively prepared by this approach using symmetric or asymmetric paracyclophanediene monomers.^35,36^

**Scheme 2:**
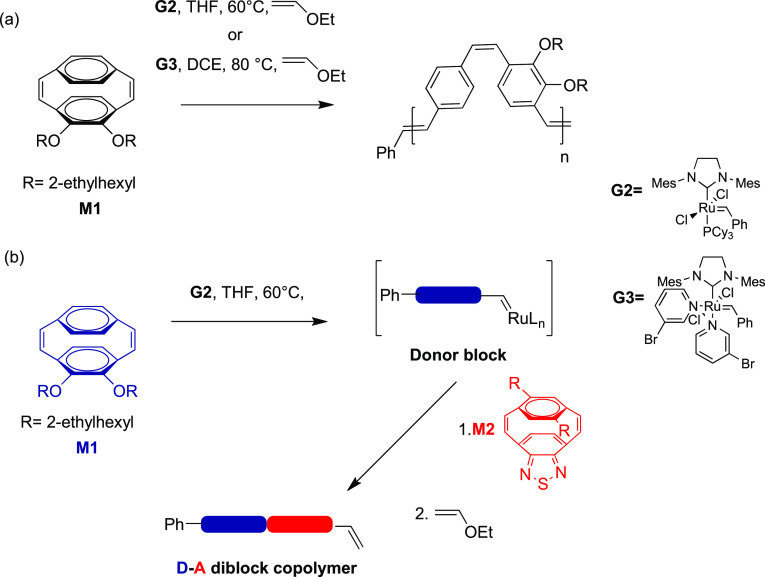
(a) ROMP of *o*-Alkoxy Monomer **M1** Using the Second- and Third-Generation Grubbs Catalysts; (b) Sequential ROMP Approach of **M1** and **M2** to D–A Diblock Arylenevinylene Copolymers

Herein, we report the preparation of well-defined 2,3-dialkoxy-substituted PPVs by the ROMP of novel 2,3-dialkoxyparacyclophanediene **M1** and the sequential ROMP with the benzothiadiazole (BT)-containing monomer **M2** to give donor–acceptor arylenevinylene diblock copolymers. The influence of the polymer microstructure on electronic as well as optical properties is reported (Scheme 2).

## Experimental Section

### Materials and General Procedure

Unless otherwise noted, all reagents were used as received from Sigma-Aldrich and Lancaster without further purification. Deuterated tetrahydrofuran (THF-*d*_8_) was degassed by a minimum of three freeze–pump–thaw cycles and stored in an argon-filled glovebox at −25 °C. The *in situ*^1^H NMR experiment using **M1** and **G2** initiators was recorded in THF-*d*_8_ using a Bruker 500 MHz spectrometer operating at either 55 or 25 °C. Longitudinal relaxation time constants (T1) for **M1** and **G2** were determined by inversion recovery, and a maximum relaxation delay of 4s was recorded, so a relaxation delay of 15s was used in all experiments. Detailed experimental procedures and characterization data for intermediates and monomer **M1** including ^1^H, ^13^C, and ^1^H–^1^H COSY NMR spectroscopy, and high-resolution mass spectrometry are given in the Supporting Information (Section S3).

### General Procedure for the *In Situ*^1^H NMR Experiment for the Reaction of **M1** with the **G2** Initiator

In an argon-filled glovebox, the **G2** catalyst was dissolved in degassed THF-*d*_8_ ([M] = 100 mM). This **G2** solution was added to the vial containing **M1**, and the vial was quickly shaken until it became homogeneous. The solution was transferred into a Young’s NMR tube, sealed, removed from the glovebox, and kept in an ice bath containing NaCl. The NMR spectrometer was then set to the desired temperature at 25 or 55 °C, and NMR spectra were recorded at 5 min intervals throughout the polymerization.

### General Procedure for the ROMP of **M1** with the **G2** Initiator

In an argon-filled glovebox, a solution of **G2** in anhydrous degassed THF was added into a vial containing monomer, **M1**([M] = 100 mM). The vial was sealed, wrapped in foil, and stirred at room temperature for 10 min. The reaction was then placed in a preheated oil bath at 60 °C and stirred until complete monomer conversion was observed by TLC and SEC. The reaction was cooled to room temperature and deoxygenated ethyl vinyl ether was added and stirred at room temperature for 4 h. The reaction was precipitated onto a short methanol/Celite column and washed with methanol, and the polymer was extracted from the Celite pad with chloroform. The chloroform was evaporated under reduced pressure to give the poly(*p*-phenylenevinylenes) as yellow amorphous films.

### General Procedure for the ROMP of **M1** with the **G3** Initiator (Microwave)

An argon-filled microwave reactor tube was charged with **M1**. The Grubbs third-generation catalyst (**G3**) was placed in an argon-filled round-bottom flask, and the catalyst was dissolved in anhydrous 1,2-dichloroethane. This catalyst solution was then injected using a syringe into the microwave tubes, and the contents were agitated to ensure a homogeneous solution. The microwave tube was then transferred to a CEM Discover Microwave Reactor and heated at a temperature of 80 °C for a period of 30 min. After this, an excess of argon-degassed ethyl vinyl ether was then injected into the microwave tubes to terminate the polymerization reaction. A small magnetic stirrer bar was added to the tubes and the mixtures were stirred overnight at room temperature using a magnetic stirrer. The polymer was then precipitated into a methanol-filled Celite plug and washed with an excess of methanol. The polymer was removed from the Celite pad by the addition of dichloromethane, and evaporation of the solvent gave the desired polymer **8d** as a glassy solid.

### Sequential ROMP of Monomers **M1** and **M2** with the **G2** Catalyst

In an argon-filled glovebox a solution of the **G2** catalyst in anhydrous, degassed THF ([M] = 100 mM) was added into a vial containing cyclophanediene monomer **M1**. The vial was sealed, wrapped in an aluminum foil, and mixed at room temperature for 10 min. The reaction mixture was placed in a preheated DrySyn aluminum block at 60 °C and stirred for 8 h until consumption of the monomer was observed (SEC and TLC). The reaction mixture was cooled to room temperature, and a solution of the second monomer **M2** in THF (0.3 mL) was added. The reaction mixture was then stirred at 60 °C for 12 h until the consumption of the monomer **M2** was confirmed by SEC and TLC, and an excess of deoxygenated ethyl vinyl ether was added, followed by stirring at room temperature for 6 h. The crude polymer was precipitated into a short methanol/Celite column, washed with methanol, and the product was extracted from the Celite with chloroform. The chloroform was removed under reduced pressure and dried to give the desired diblock copolymer as a brown amorphous film.

### General Procedure for Photoisomerization of Polymers

Polymers **8a–c**, **8d**, and **9** were dissolved in degassed dichloromethane (1 mg/mL) in an argon-filled glovebox. The vial was sealed, removed from the glovebox, and subjected to photoisomerization by irradiation using a UV lamp (λ = 365 nm) for 48 h. After evaporation of the solvent, the polymers were isolated as a yellow solid, *trans***8a–c**, **8d**, and brown solid, **9**, in quantitative yields.

## Results and Discussion

**Scheme 3:**
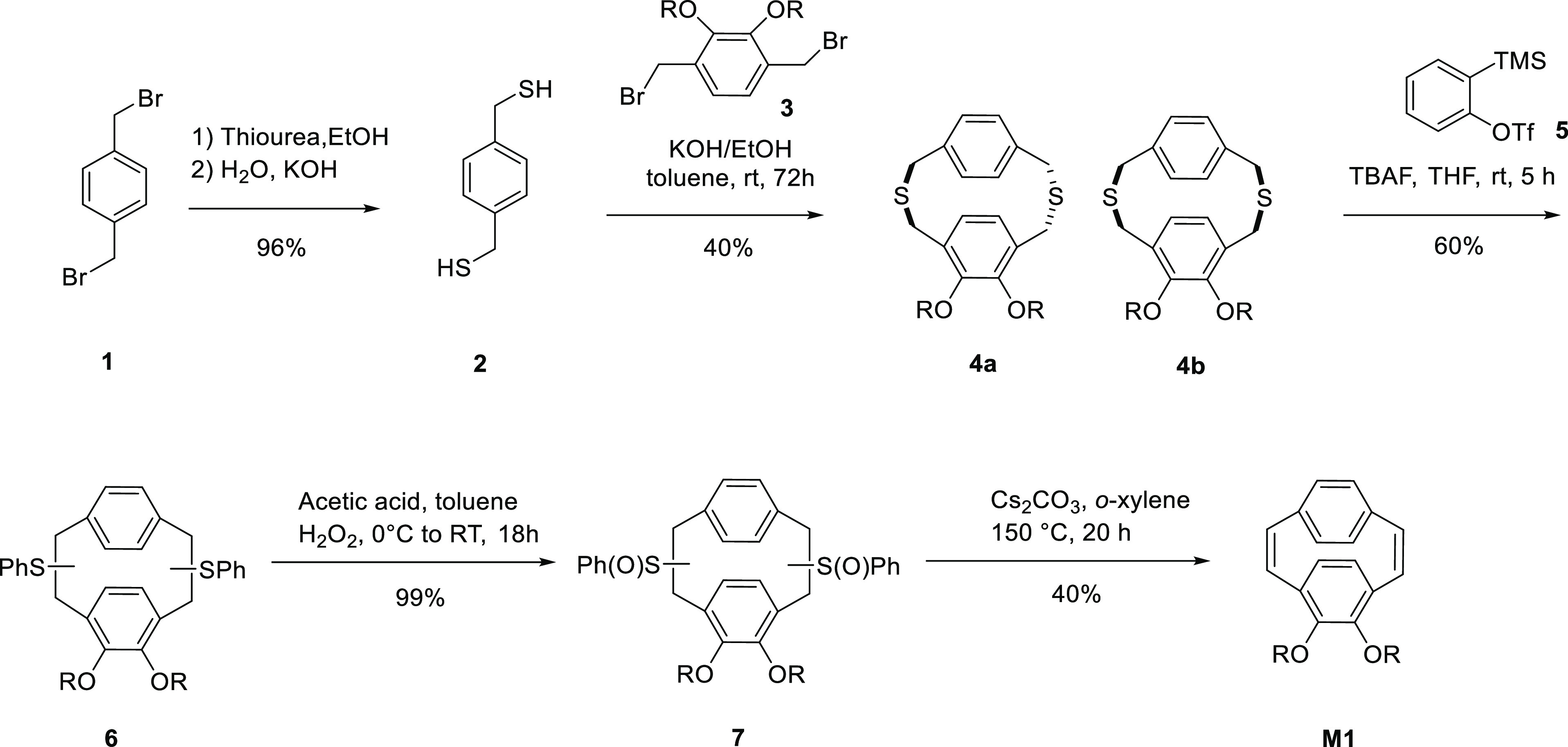
Synthesis of Dialkoxy Paracyclophanediene Monomer **M1**

The 4,5-diethylhexyloxy-[2.2]paracyclophane-1,9-diene monomer **M1** was synthesized as shown in Scheme 3. Dithiacyclophanes **4a** and **4b** were prepared by the slow addition of a toluene solution containing equimolar amounts of compounds **2** and **3** to a large volume of ethanol containing potassium hydroxide. This reaction gave the desired dithiacyclophanes as a mixture of two conformers, chair **4a** and boat **4b**. Dithiaparacyclophane chair **4a** and boat **4b** were characterized using GC–MS analysis, showing a peak with a molecular weight of 528 (M^+^). In the boat conformer **4b**, the unsubstituted aromatic ring results in only one singlet at 7.01 ppm, integrating four equivalent hydrogens (**H**^**2**^) (Figure S2). This confirms that a large degree of ring flipping is possible in this conformer.^41^ According to the crystal structure of analogous compounds in the chair conformer, the rings are more eclipsed, while the rings are more staggered in the boat conformer.^41^ The hydrogen resonances of the substituted aromatic ring of this conformer **4b** appear as a singlet peak of 6.54 ppm integrating two hydrogens (**H**^**1**^). The four diastereotopic hydrogens of the methylene groups adjacent to the oxygens result in the multiplet peak at 3.71–3.79 ppm (ABX system, 4dd), integrating to four hydrogens. The thioether environments in **4b** are split into two singlets at 3.60 (**H**^**3**^) and 3.56 ppm (**H**^**4**^), integrating to four hydrogens each.

A benzyne-induced Stevens rearrangement was performed by the dropwise addition of a THF solution of tetrabutylammonium fluoride (TBAF·3H_2_O) to a mixture of compounds **4** and **5** dissolved in THF at room temperature. This ring contraction reaction yielded a complex mixture of regio- and stereo-isomers of the *bis*-phenyl sulfide compound **6**. These isomers are readily oxidized to the corresponding *bis*-sulfoxides **7** using hydrogen peroxide as the oxidant. The final conversion to the desired 4,5-diethylhexyloxy-[2.2]paracyclophane-1,9-diene **M1** was achieved by the thermal elimination of phenylsulfinic acid in the presence of a base, cesium carbonate. A peak at 461 *m*/*z* in the APCI-MS spectrum confirmed the successful preparation of **M1**; full characterization details for **M1** are given in the Supporting Information.

**Figure 1:**
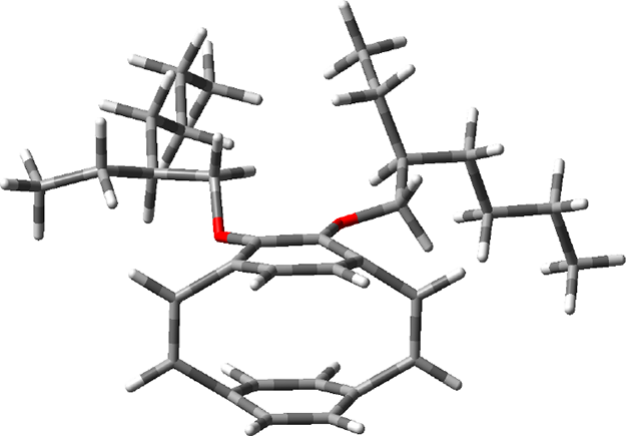
DFT-optimized geometry of **M1**.

The monomer is a viscous liquid and was isolated as a diastereomeric mixture due to the planar chirality of the substituted aromatic ring and the stereocenters of the branched ethylhexyloxy side chains. It was not possible to obtain single crystals suitable for X-ray crystallography, but a preliminary insight into the strained nature of this monomer was obtained by density functional theory (DFT) calculations of the optimized ground-state geometry using B3LYP/6-311G(d,p) (Figure 1). In the calculated structure, the vinylene bond length is 1.34 Å, which is in agreement with that of a standard *cis*-vinylene bond of 1.32 Å.^42^ The π–π distance between the ring systems is 3.06 Å and the torsion angles of the *cis* vinyl part of **M1** are 2.30 and 2.33°. The ring strain of the optimized geometry was calculated to be 76.1 kcal·mol^–1^, which is large enough to lead to efficient ROMP, as observed for other ring systems (e.g., the ring strain of norbornene is 23.9 kcal mol^–1^).^43^

The ROMP of monomer **M1** using the **G2** initiator was examined by an *in situ*^1^H NMR experiment in *d*_8_-THF conducted at 55 °C in a Young’s NMR tube. In this experiment, complete monomer consumption was observed after 36 h (see Figure S11); by contrast, bulk reactions using **G2** conducted in stirred THF solutions at 60 °C were generally complete within 8 h. In the carbene region of the ^1^H NMR spectrum, the carbene chain ends A and B corresponded to the coordination of PCy_3_ to the ruthenium center and are assigned to the signals at higher chemical shifts. The signal observed at 19.17 ppm is assigned to chain end B with the substituted benzylidene (H_b_) and the signal at 18.34 to chain end A with the unsubstituted benzylidene (H_a_). The signal at 15.35 ppm is assigned to carbene species C with a lower chemical shift owing to the coordination of the oxygen atom in the ortho-position of the benzylidene ring to the ruthenium which leads to a significant shielding of the chemical shift. The living character of the polymerization process was shown by the linear correlation between −ln([*M*_t_]/[*M*_0_]) and time (Figure S11c) indicative of the first-order consumption of monomer **M1**. The slope of this plot at 55 °C gave an apparent rate constant for propagation, *k*_p_^app^ of 0.0009 min^–1^; this is smaller than that observed for the polymerization of the analogous 2,5-dialkoxyparacyclophanedienes using initiator **G2** at 40 °C (*k*_p_^app^ = 0.0026 min^–1^). The lower propagation rate constant is presumably due to the stronger Ru–O interactions in the active ruthenium carbene complex formed with the two orthoalkoxy substituents of monomer **M1**.^44^

Monomer **M1** can also be polymerized by the third-generation Grubbs catalyst using anhydrous 1,2-dichloroethane (DCE) as the solvent. This catalyst is known to initiate ROMP more rapidly than **G2**, and this reaction mixture was heated in a microwave to 80 °C to accelerate the reaction and shorten the reaction time. By taking small aliquots of the reaction mixture and running SEC, it was found that after 30 min, the target molecular weight had been achieved and monomer conversion was close to complete. The polymerization was quenched by the addition of an excess of ethyl vinyl ether and then purified by precipitation using MeOH over Celite and recovered by elution with chloroform.

**Figure 2:**
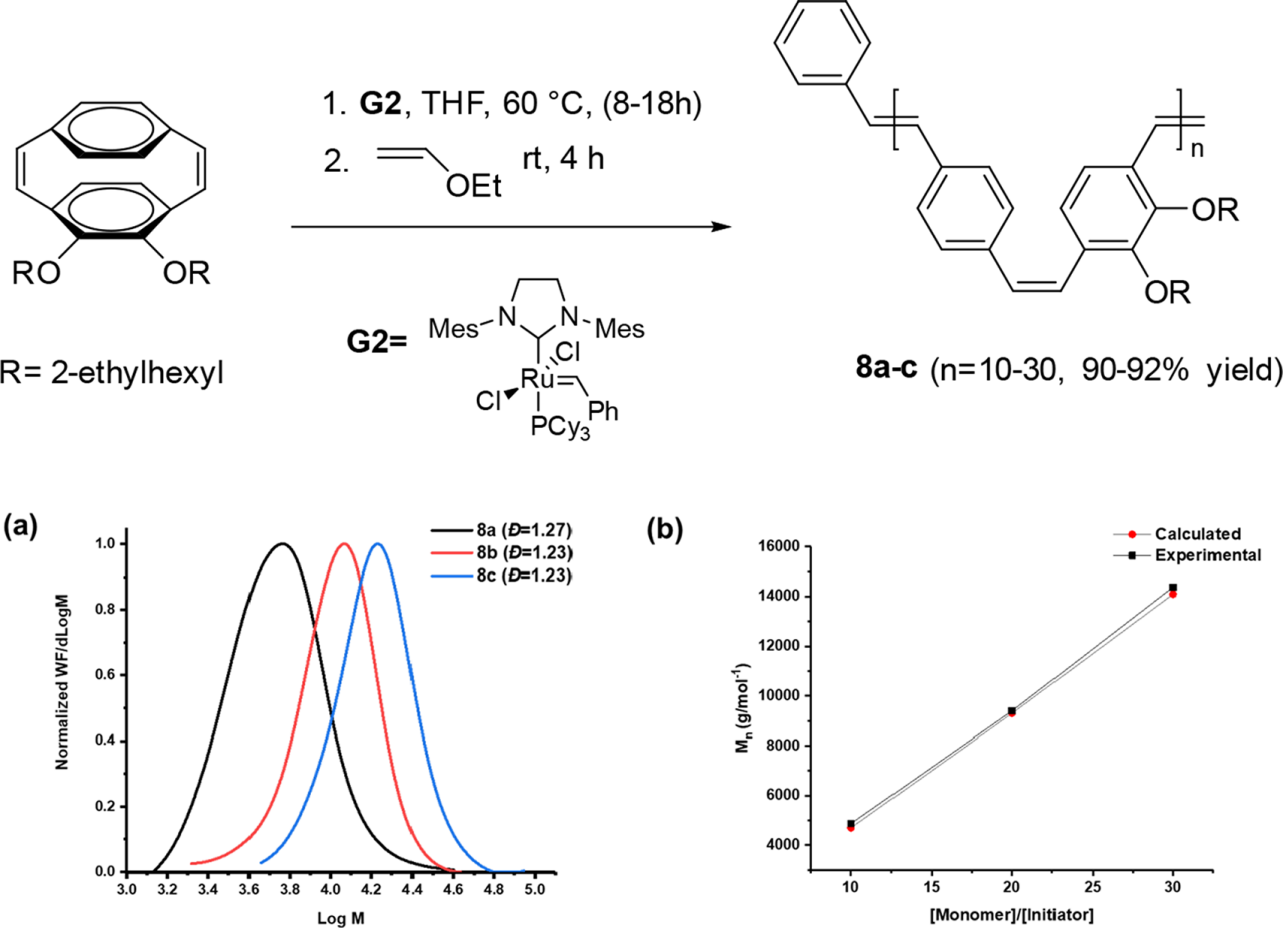
ROMP of monomers **M1** with the **G2** catalyst. (a) Molecular weight distribution of polymers **8a–c** (SEC in THF) and (b) Dependence of *M*_n_ of the polymers **8a–c** on the [**M**]/[**G2**] ratio.

The number-average molecular weight (*M*_n_) of the PPVs (**8a–c**) isolated from the reaction with **G2** in stirred solutions were measured by SEC and the observed *M*_n_ showed a linear dependence on the initial [M]/[I] ratio with a correlation coefficient of 0.999, confirming the good control achieved in this polymerization, dispersities were in the range of 1.23–1.30 and yields of recovered polymer were over 90% (Figure 2, Table1).

**Table 1:** SEC Data of Polymers

					SEC data purified^d^			
entry	PPV	[monomer]/[initiator]	*T* (°C)	t	*M*_n(calc)_ (kDa)	*M*_n(obs)_ (kDa)	*Đ*	% yield
1^a^	**8a**	[**M1**]/[**G2**] = 10	60	36 h	4.7	5.1	1.30	95
2^b^	**8a**	[**M1**]/[**G2**] = 10	60	8 h	4.7	4.9	1.27	91
3^b^	**8b**	[**M1**]/[**G2**] = 20	60	12 h	9.3	9.4	1.23	90
4^b^	**8c**	[**M1**]/[**G2**] = 30	60	18 h	14.1	14.9	1.23	92
5^c^	**8d**	[**M1**]/[**G3**] = 10	80	30 min	4.7	4.7	1.4	90

a *In situ*^1^H NMR experiments in THF-*d*_8_ at 55 °C.

b Reactions were performed in a screw-cap vial using degassed anhydrous THF as the solvent at 60 °C.

c Reaction was performed in a microwave vial using degassed anhydrous 1,2-dichloroethane as the solvent at 80 °C.

d *M*_n(calc.)_ values were calculated from **[M1]**/**[G2]** and **[M1]**/**[G3]** ratios, and *M*_n(obs.)_ values were measured by SEC against narrow molecular-weight polystyrene standards.

**Figure 3:**
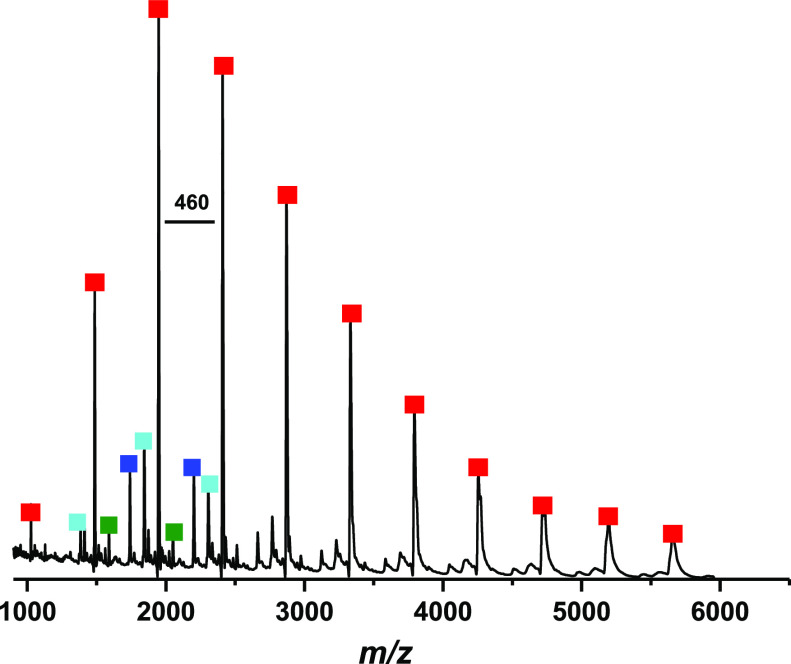
MALDI-TOF mass spectrum of polymer **8a** with an expected degree of polymerization of 10 repeat units.

Matrix-assisted laser desorption/ionization time-of-flight mass spectrometry (MALDI-TOF-MS) of polymer **8a** (*n* = 10) shows a major series of peaks at a high molecular weight between 1000 and 6000 *m*/*z*. All of these peaks (red ■) are separated by an interval of 460 Da that corresponds to the molecular weight of the added monomer **M1** (Figure 3). This series of peaks suggests that the polymers are terminated with vinyl and phenyl end groups as expected. The lower intensity series of peaks that are separated by 460 Da (turquoise ■), (green ■), and (blue ■) are associated with intrachain metathesis or back-biting of the active chain end and consist of either cyclic or linear PPVs with one additional phenylenevinylene or disubstituted phenylene vinylene ring. The structures of these species are shown in Figure S23.

**Figure 4:**
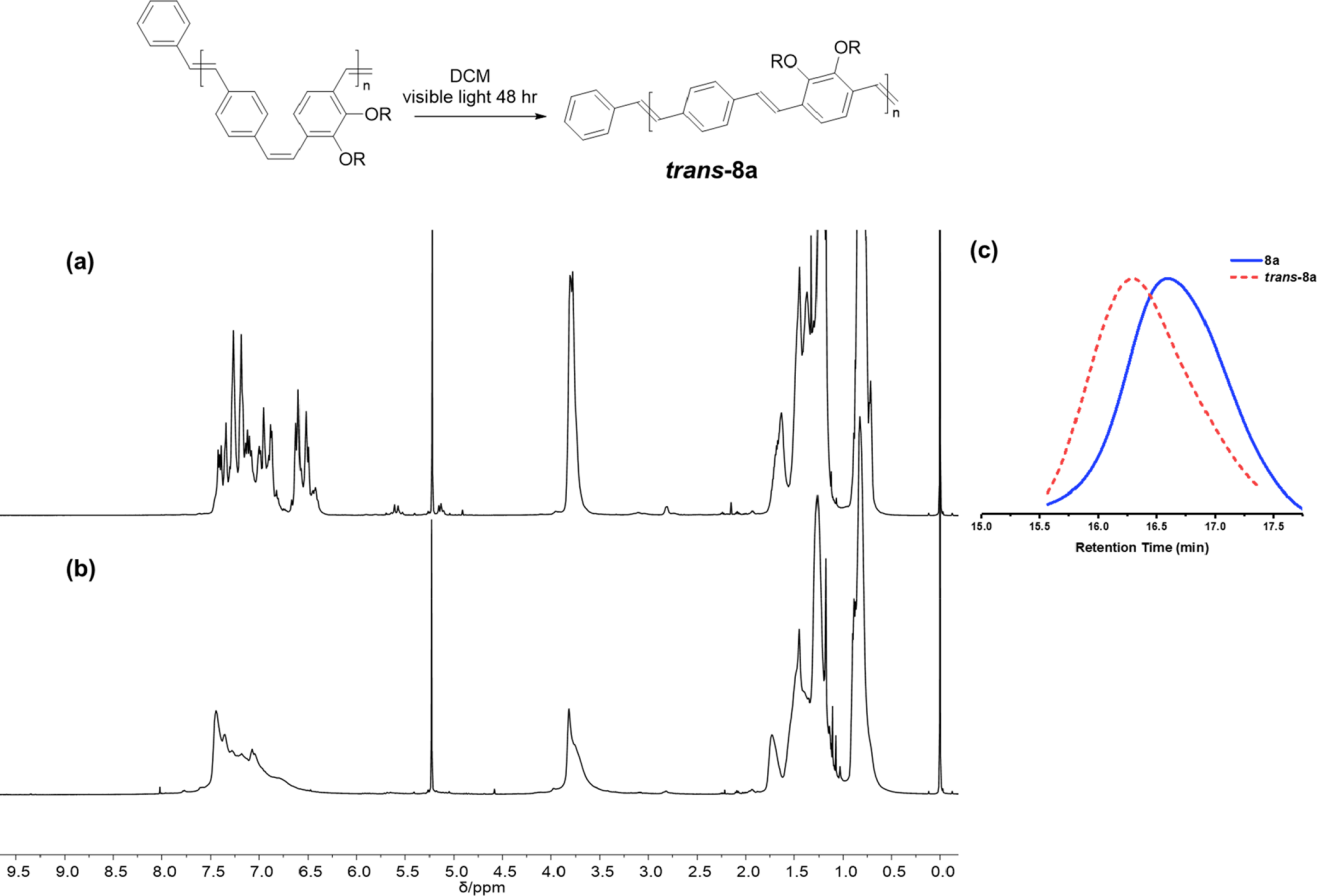
Photoisomerization of the **8a** (a) ^1^H NMR spectrum of *cis*/*trans*-vinylene polymer **8a** in CD_2_Cl_2_ (b) *trans*-**8a** in CD_2_Cl_2_ (c) SEC traces of **8a** and *trans*-**8a** in THF.

The ^1^H NMR spectra (see Figure 4) confirmed that the isolated polymers have alternating *cis*/*trans*-vinylene stereochemistry due to the metathesis of only one vinylene bond of **M1**. The peak at δ 3.79 ppm can be assigned to the hydrogens of the methylene groups attached to the oxygen for both the *trans*- and *cis*-vinylene linkages. This contrasts strongly with the observed ^1^H NMR spectrum of the analogous 2,5-dialkoxyphenylene vinylene polymers. In this case, the signals for the methylene groups of the alkoxy substituents adjacent to the *cis*-vinylenes are usually observed at δ 3.5 ppm and those adjacent to the *trans*-vinylenes at *ca*. δ 4.0 ppm.^45^ The difference is presumably due to the restricted conformations imposed by the ortho placement of the alkoxy substituents in polymer **8**. Signals for *cis*-vinylene groups appear between 6.34 to 6.68 ppm and those for the *trans*-vinylene and other aromatic hydrogens appear further downfield after 6.70 ppm. Comparing the relative areas of the peaks at 3.79 ppm for the alkoxy methylene hydrogens and those at 6.34–6.68 ppm for those of the *cis*-vinylenes shows that these are in the expected 1:0.5 ratio. Absolute values for the degree of polymerization were determined by integration of the signals for the vinyl end groups observed at 5.16 and 5.60 ppm against those for the methylene groups attached to oxygen. These values are in agreement with the calculated values from [**M**]/[**I**] ratios (Table S1).

Complete isomerization to all-*trans***8a** was conducted in a dilute solution (1 mg/mL in degassed dichloromethane) under exposure to visible light. The ^1^H NMR spectrum recorded at the end of the reaction showed that the signals between 6.34 and 6.68 ppm associated with the presence of *cis*-vinylenes completely disappear after isomerization (Figure 4). In general, the signals for the *trans* vinylene and aromatic signals are simplified after isomerization. In SEC analysis, the all-*trans* isomers have lower retention times compared to the analogous *cis/trans* forms due to the higher hydrodynamic volume of the trans isomer.

**Figure 5:**
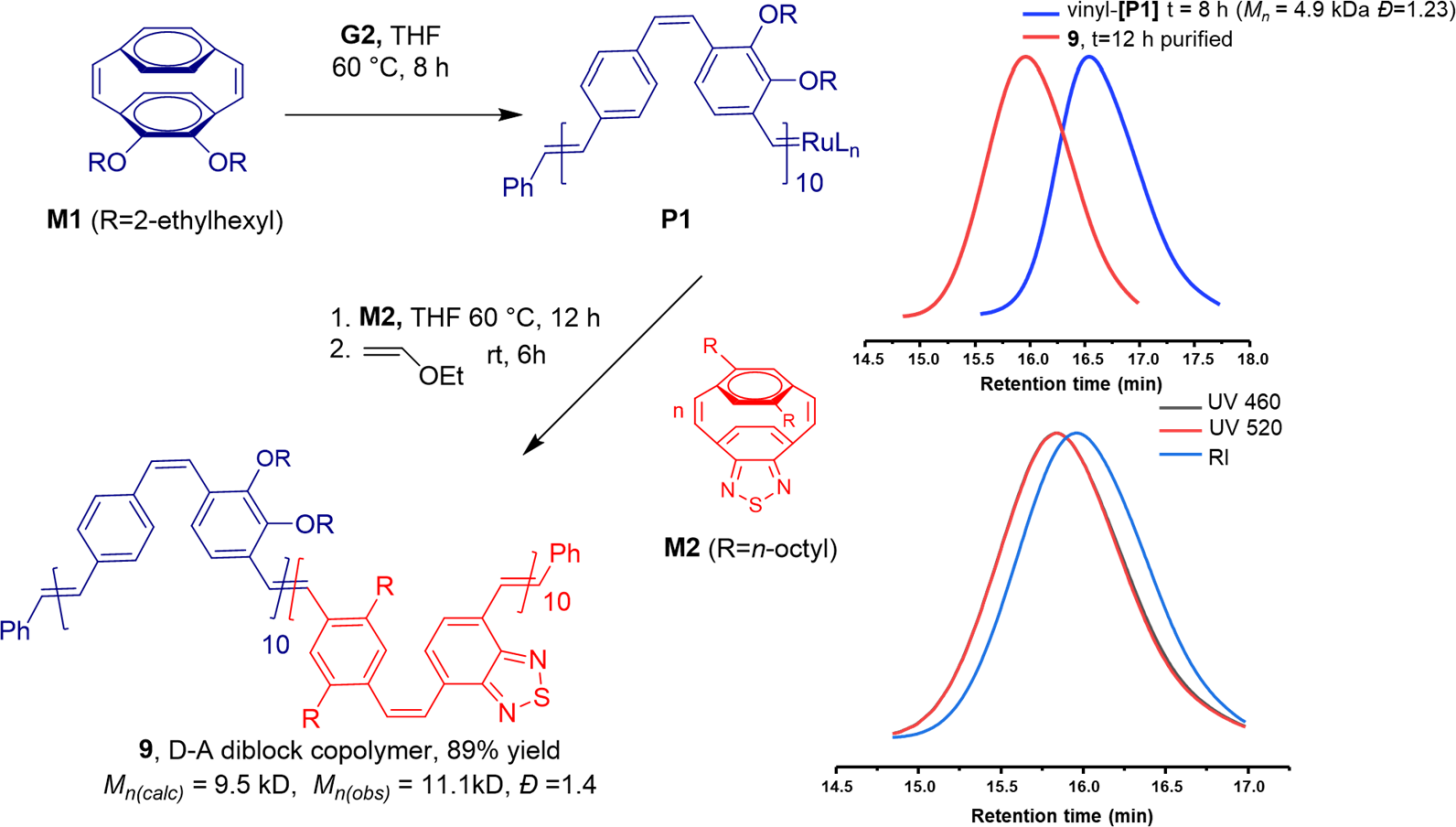
Synthesis of the D–A diblock copolymer via the sequential ROMP of cyclophanediene monomers **M1** and **M2** (SEC in THF).

The living nature of the ROMP of **M1** using the **G2** initiator was confirmed by the preparation of a block copolymer of **M1** and **M2** by a sequential ROMP. This reaction was performed in degassed THF at 60 °C in an argon-filled glovebox, and the initial conversion of monomer **M1** (**[M1]**/**[G2]** = 10) was monitored by TLC and SEC. After complete consumption of **M1**, monomer **M2** (**[M2]**/**[G2]** = 10) was added and the reaction mixture was stirred at 60 °C for 12 h. The reaction was finally quenched by the addition of excess ethyl vinyl ether at ambient temperature to afford D–A diblock copolymer **9** in an 89% isolated yield after purification (*M*_n(obs)_ = 11.1 kDa and *Đ* = 1.40) (Figure 5).

The ^1^H NMR spectrum of the block copolymer **9** (Figure S16) confirmed the efficient incorporation of monomers **M1** and **M2**, as evidenced by the integration of 1:1 for the signals at δ 3.79 ppm corresponding to the alkoxy methylenes of monomer **M1** to that between 2.04 and 2.97 ppm corresponding to the benzylic methylenes of monomer **M2**. A comparison of the SEC traces recorded after complete consumption of **M1** and for the isolated block copolymer **9** shows the efficient chain extension during the sequential ROMP. The SEC of **9** was recorded using both RI and UV–vis detection. The traces associated with recording at the absorption maxima of the **M1**- and **M2**-derived blocks (460 and 520 nm) are essentially superimposable, confirming the formation of the diblock copolymer **9** with no evidence of homopolymers derived from **M1** or **M2** (Figure 5).

**Figure 6:**
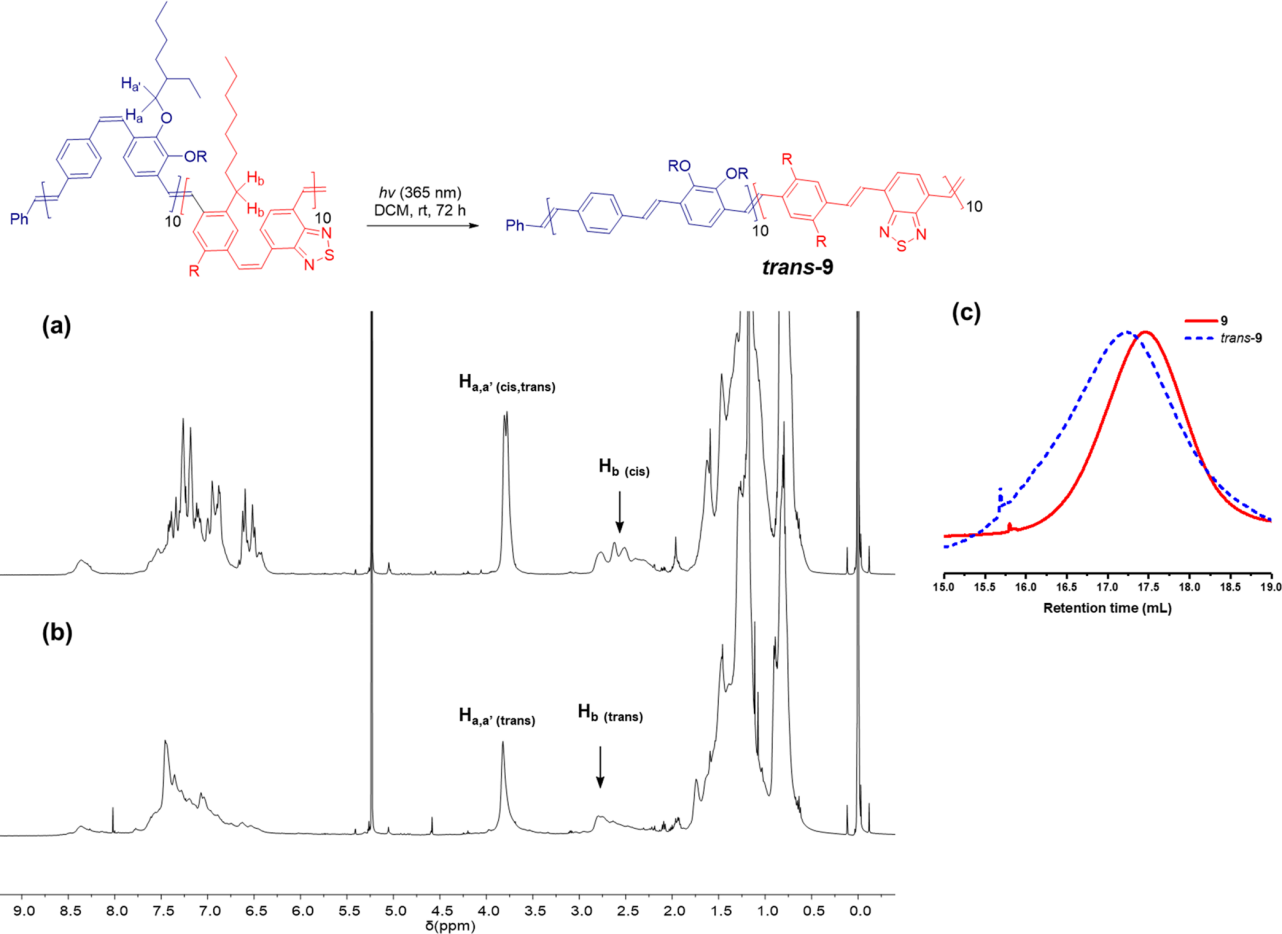
Photoisomerization of D–A diblock copolymer **9** from *cis/trans* to *trans* alkene stereochemistry: (a) ^1^H NMR spectra of **9**, (b) *trans*-**9** in DCM-*d*_2_, and (c) SEC traces of **9** and *trans*-**9** in CHCl_3_.

The *cis/trans* block polymer **9** can also be isomerized to *trans*-**9** by dissolving the polymer in a solution of argon-degassed DCM and exposing it to UV light at a wavelength of 365 nm for a period of 48 h (see Figure 6). The disappearance of the signals between 6.34 and 6.68 ppm associated with the *cis*-vinylene unit and the phenyl groups neighboring to *cis*-vinylene groups confirmed the isomerization to an all-*trans* vinylene configuration.

**Figure 7:**
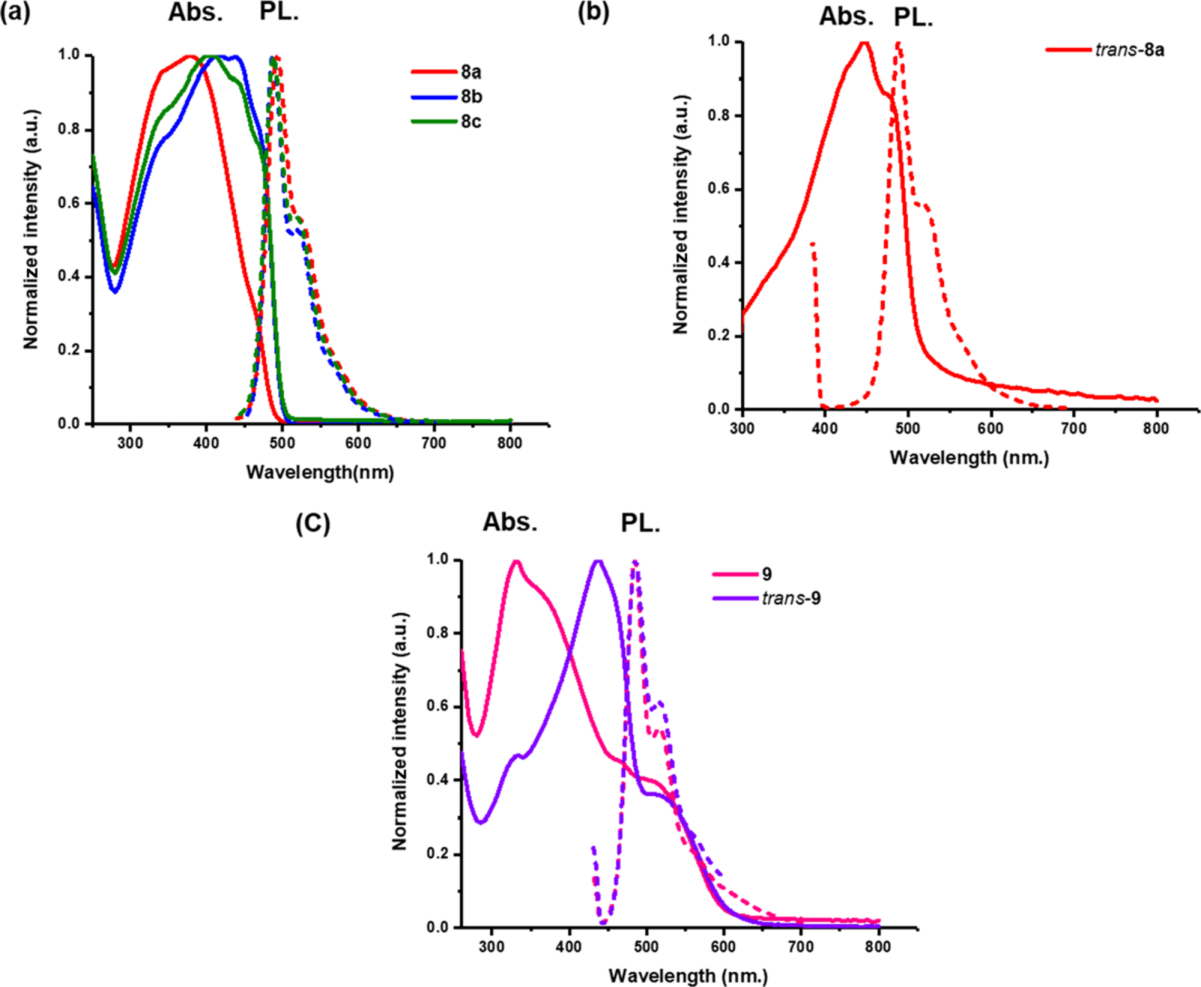
Absorption and emission profiles of copolymers (a) **8a–c** (Ex = 380 nm), (b) *trans***8a** (Ex = 430 nm), and (c) block copolymer **9** (Ex = 330 nm) and *trans*-**9** (Ex = 430 nm) in CHCl_3_.

The optical properties of the polymers were measured in dilute solutions of chloroform and as thin films (Figure 7 and Table 2). All polymers showed broad absorption bands with comparable absorption maxima (λ_max_) values in solution and the solid state. Polymer **8a** exhibited a λ_max_ of 384 nm in the chloroform solution compared to a λ_max_ of 438 nm for the *trans*-isomer and a red shift of 54 nm. In the film, the same trends were observed as were seen in the solution. The *cis–trans* polymer **8a** (*n* = 10) exhibited a λ_max_ of 386 nm, while the *trans* polymer **8a** (*n* = 10) exhibited a λ_max_ of 446 nm. The absorption spectra of polymers **8b** and **8c** (*n* = 20 and *n* = 30) shifted to longer wavelength when compared to **8a** (*n* = 10), consistent with more extended conjugation over the longer chain lengths (see Figure 7 and Table 2). By contrast, the photoluminescence spectra recorded for these polymers showed nearly identical emissions around 490 nm in solution and 500 nm in a thin film for all of the chain lengths due to emission from the most extended conformation of the polymer chain. There was a small bathochromic shift in the emission spectra of the *trans*-polymer to *ca.* 500 nm when excited at 430 nm.

**Table 2:** Optical Properties of the Polymers^d^

	λ_(abs. max)_ (nm)^a^		λ_(exc.)_ (nm)		λ_(em_. _m__ax)_ (nm)			
	solution	film	solution	film	solution	film	Φ_PL_ (%)^b^	E_g (opt.)_ (eV)^c^
**8a**	384	386	380	380	490(546)	504(539)	0.64	2.50
**8b**	410	414	380	380	488(530)	505(536)	0.53	2.49
**8c**	393	398	380	380	488(542)	505(539)	0.70	2.49
**8d**	376	383(475)	380	380	484	504(539)	0.63	2.51
**8a**^e^	438	446	430	430	498(548)	506(540)	0.18	2.43
**9**	340, 505	334, 508	330	490	484(518)	642	0.14	2.20
**9**^e^	436, 532	438, 540	430	520	485(518)	658	0.19	2.01

a Measurements recorded in dilute solutions of chloroform.

b Measured in an integrating sphere.

c *E*_g_ = 1240/λ_onset_.

d HOMO = (*E*_ox_^onset^ – Fc_ox_) + 4.8 eV, LUMO = (*E*_red_^onset^ – Fc_ox_) + 4.8 eV_._

e *E*_g (elc.)_ = HOMO – LUMO.

Analogous 2,3-dialkoxyphenylene-substituted PPVs have been previously prepared by the Gilch methodology.^31^ However, in these polymers, every phenylene ring is functionalized with two alkoxy groups in the ortho-positions and the vinylenes are in a *trans*-stereochemistry. The absorption maxima of the butoxy-substituted polymers in a thin film were reported to be 454 nm; these absorptions were significantly blue-shifted relative to the thin film spectra reported for the 2,5-substituted MEH-PPV at 500 nm.^31^ The emission spectra of polymers **8a–c** were blue-shifted when compared to those of the analogous *cis*,*trans*-2,5-dialkoxy PPVs prepared by ROMP (λ_max_ = 527 nm in a dilute dichloromethane solution).^45^ The emission of the *trans*-2,3-dibutoxyphenylene PPV previously reported, measured as a thin film, was 519 nm^31^ which is slightly red-shifted over the polymers prepared by ROMP due to having each phenylene ring substituted with alkoxy groups. The photoluminescence quantum yields obtained for the 2,3-dialkoxy polymers are higher than for 2,5-alkoxy polymers. For 2,5-alkoxy polymers, these ranged from 0.22 to 0.31.^45^ For polymers **8a–8c**, the highest quantum yield was observed for the *cis/transn* = 30 polymer, giving a value of 0.70 with the lowest value being attributed to the *trans***8a** polymer (0.18).

The absorption spectra of the block copolymer **9** were also recorded in dilute chloroform solutions and as thin films. The spectrum of the isolated *cis/trans* polymer is a weighted average of the absorptions of the two constituent blocks with the major absorption centered around 340 nm associated with the absorption of the 2,3-dialkoxy phenylene block and the longer wavelength absorption at 500 nm due to the BT-containing block.^37^ Excitation of **9** at 330 nm in solution resulted in emission at 484 nm which corresponds to the emission maximum of the donor block. In the thin film, excitation of **9** at 490 nm or *trans*-**9** at 520 nm led to a highly red-shifted emission at 642 and 658 nm, respectively, consistent with emission from the block derived from **M2**(37) (see Figures S27 and S28, Table S2). Reduced optical band gaps were measured for all the *trans* polymers due to the extended conjugation in this isomer. The fluorescence quantum yields (Φ_PL_) of the *cis*/*trans***9** and *trans***9** are 0.14 and 0.19, respectively. The *trans*-polymer **9** shows higher quantum efficiencies as the interchain energy transfer in the *cis/trans* isomers is more effective than that in the *trans*-polymer **9**, and the *cis/trans* polymer **9** undergoes isomerization of the *cis* alkene upon illumination.^46^

**Figure 8:**
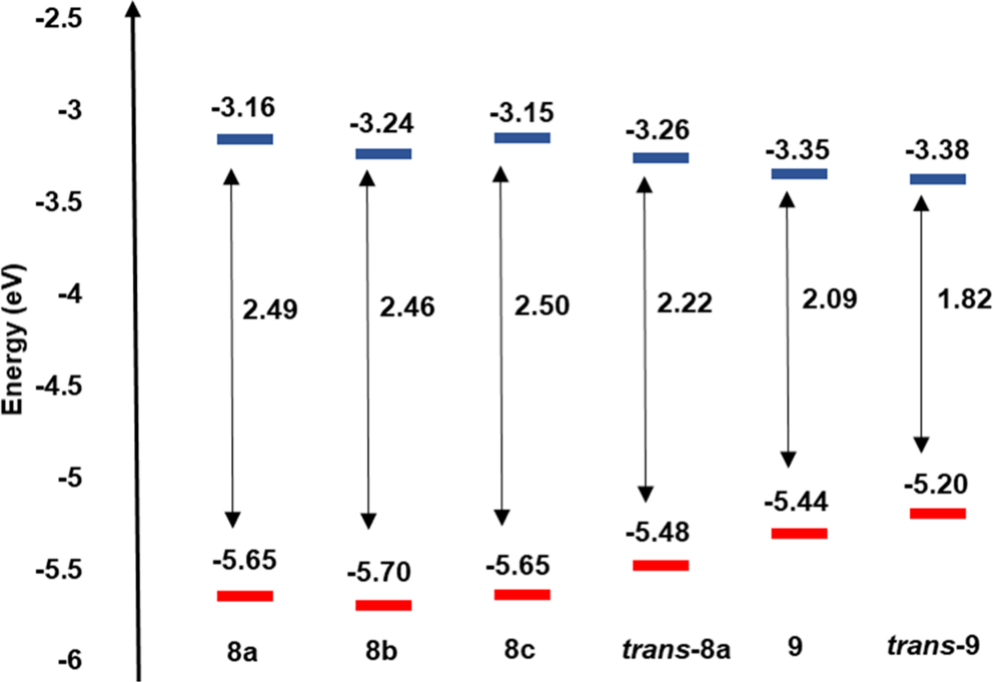
Electrochemical HOMO and LUMO energy levels of the polymers.

The electrochemical properties of polymers were studied by cyclic voltammetry in the solid state using tetrabutylammonium hexafluoride in acetonitrile (0.1 M) as the electrolyte, by depositing the polymers on a platinum foil working electrode. The calculated HOMO and LUMO energy levels for the *cis*/*trans***8a**–**8c** are approximately −5.65 and −3.16 eV, respectively. For the corresponding all-*trans***8a**, the HOMO and LUMO values are −5.48 and −3.26 eV, respectively (Figure 8 and Table S3). There is a small difference between the electrochemical band gap (*ca.* 0.27 eV) of the *trans***8a** and that of the *cis/trans***8a**, which is consistent with an increase in the conjugation length of the *trans* polymer. The calculated HOMO and LUMO energy levels for *cis*/*trans***9** are approximately −5.44 and −3.35 eV, respectively. For the all-*trans***9**, the HOMO value is −5.20 eV and the LUMO value is −3.38 eV. Isomerization of **9** to the all-*trans* polymer **9** again leads to a reduction in the band gap owing to the extended conjugation of the *trans* PPV configuration. The band gaps for D–A block copolymers **9** and *trans*-**9** are lower than those of all homopolymers **8a–8c**, indicating that the incorporation of an acceptor BT unit in the block copolymer effectively reduces the band gap.

**Figure 9:**
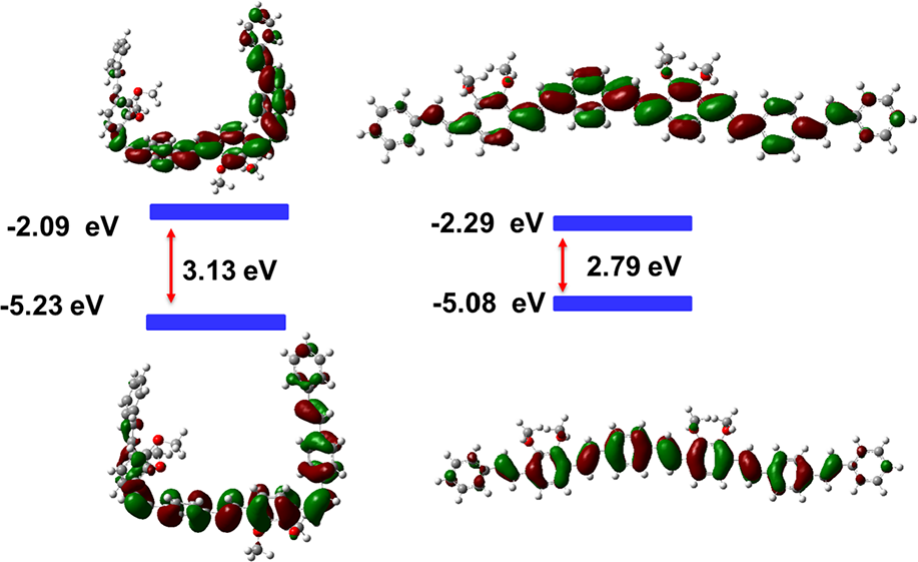
Frontier orbitals for TD-DFT calculation using B3LYP/6-311G(d,p) of a dimer of *cis*/*trans***8** and all-*trans***8**.

**Figure 10:**
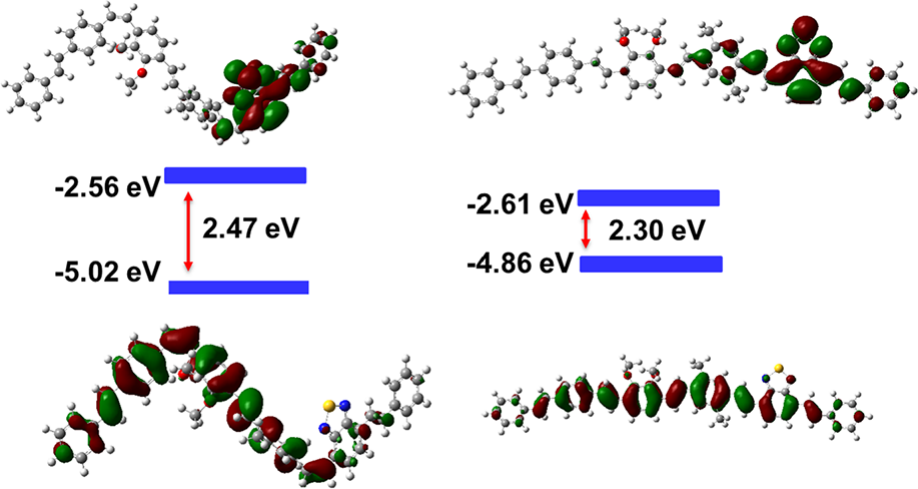
Frontier orbitals for TD-DFT calculation using B3LYP/6-311G(d,p) of a dimer of *cis*/*trans***9** and all-*trans* D–A diblock copolymer **9**.

To examine the electronic states of these polymers, the molecular orbitals were calculated using DFT. The diethylhexyl side chains of all the structures were replaced with methyl groups to reduce computational costs without influencing the energy of the HOMO and LUMO. The contour plots of the HOMO and LUMO by B3LYP/6-311G(d,p) for a segment of **8** and **9** are shown in Figures 9 and 10, respectively. For polymer **8**, the frontier molecular orbitals are delocalized over the whole π-conjugated backbone and the HOMO and LUMO with those of the all-*trans* isomer more extensively delocalized than those of the *cis/trans* isomer. For polymer **9**, the HOMO is delocalized on both the electron-rich dialkoxyphenyl and electron-deficient BT unit and the LUMO is localized on the electron-withdrawing BT unit, as expected.^37^ In general, the energy gaps of the all-*trans* polymers are lower than the parent *cis/trans* isomers, calculated in the gas phase due to the extended conjugation of *trans*-vinylene.

## Conclusions

In summary, the ROMP of the strained *o*-dialkoxy paracyclophanediene **M1** gives alternating phenylenevinylene copolymers and block copolymers in a well-controlled chain-growth polymerization. The linear relationship between the polymer molecular weight and [monomer]/[initiator] ratio confirmed the living nature of the polymerization. The initially obtained alternating *cis*/*trans* vinylene polymers can be photochemically isomerized to the all-*trans*-vinylene under visible light. Examination of the optical and electrochemical properties of the polymers showed that all-*trans* co-/block copolymers showed red-shifted absorption maxima with smaller optical and electrochemical band gaps owing to extended conjugation.

## Supporting Information Available

Detailed experimental procedures including spectral data for all the synthetic intermediates and polymers (PDF)

## References

[ref1] Thompson B. C.; Fréchet J. M. J. Polymer–Fullerene Composite Solar Cells. Angew. Chem., Int. Ed. 2008, 47, 58–77. 10.1002/anie.200702506. 18041798

[ref2] Dou L.; Liu Y.; Hong Z.; Li G.; Yang Y. Low-Bandgap Near-IR Conjugated Polymers/Molecules for Organic Electronics. Chem. Rev. 2015, 115, 12633–12665. 10.1021/acs.chemrev.5b00165. 26287387

[ref3] He Y.; Hong W.; Li Y. New Building Blocks for π-Conjugated Polymer Semiconductors for Organic Thin Film Transistors and Photovoltaics. J. Mater. Chem. C 2014, 2, 8651–8661. 10.1039/c4tc01201a.

[ref4] Henson Z. B.; Müllen K.; Bazan G. C. Design Strategies for Organic Semiconductors beyond the Molecular Formula. Nat. Chem. 2012, 4, 699–704. 10.1038/nchem.1422. 22914189

[ref5] Beaujuge P. M.; Fréchet J. M. J. Molecular Design and Ordering Effects in π-Functional Materials for Transistor and Solar Cell Applications. J. Am. Chem. Soc. 2011, 133, 20009–20029. 10.1021/ja2073643. 21999757

[ref6] Zhong C.; Duan C.; Huang F.; Wu H.; Cao Y. Materials and Devices toward Fully Solution Processable Organic Light-Emitting Diodes. Chem. Mater. 2011, 23, 326–340. 10.1021/cm101937p.

[ref7] Young C. A.; Hammack A.; Lee H. J.; Jia H.; Yu T.; Marquez M. D.; Jamison A. C.; Gnade B. E.; Lee T. R. Poly(1,4-Phenylene Vinylene) Derivatives with Ether Substituents to Improve Polymer Solubility for Use in Organic Light-Emitting Diode Devices. ACS Omega 2019, 4, 22332–22344. 10.1021/acsomega.9b02396. 31909316 PMC6941186

[ref8] Coughlin J. E.; Henson Z. B.; Welch G. C.; Bazan G. C. Design and Synthesis of Molecular Donors for Solution-Processed High-Efficiency Organic Solar Cells. Acc. Chem. Res. 2014, 47, 257–270. 10.1021/ar400136b. 23984626

[ref9] Wang C.; Dong H.; Hu W.; Liu Y.; Zhu D. Semiconducting π-Conjugated Systems in Field-Effect Transistors: A Material Odyssey of Organic Electronics. Chem. Rev. 2012, 112, 2208–2267. 10.1021/cr100380z. 22111507

[ref10] Duarte A.; Pu K.-Y.; Liu B.; Bazan G. C. Recent Advances in Conjugated Polyelectrolytes for Emerging Optoelectronic Applications. Chem. Mater. 2011, 23, 501–515. 10.1021/cm102196t.

[ref11] Kraft A.; Grimsdale A. C.; Holmes A. B. Electroluminescent Conjugated Polymers—Seeing Polymers in a New Light. Angew. Chem., Int. Ed. 1998, 37, 402–428. 10.1002/(sici)1521-3773(19980302)37:4<402::aid-anie402>3.0.co;2-9. 29711177

[ref12] Grimsdale A. C.; Leok Chan K.; Martin R. E.; Jokisz P. G.; Holmes A. B. Synthesis of Light-Emitting Conjugated Polymers for Applications in Electroluminescent Devices. Chem. Rev. 2009, 109, 897–1091. 10.1021/cr000013v. 19228015

[ref13] Burroughes J. H.; Bradley D. D. C.; Brown A. R.; Marks R. N.; Mackay K.; Friend R. H.; Burns P. L.; Holmes A. B. Light-Emitting Diodes Based on Conjugated Polymers. Nature 1990, 347, 539–541. 10.1038/347539a0.

[ref14] Zheng H.; Zheng Y.; Liu N.; Ai N.; Wang Q.; Wu S.; Zhou J.; Hu D.; Yu S.; Han S.; Xu W.; Luo C.; Meng Y.; Jiang Z.; Chen Y.; Li D.; Huang F.; Wang J.; Peng J.; Cao Y. All-Solution Processed Polymer Light-Emitting Diode Displays. Nat. Commun. 2013, 4, 1971 10.1038/ncomms2971. 23736123

[ref15] King J. T.; Granick S. Operating Organic Light-Emitting Diodes Imaged by Super-Resolution Spectroscopy. Nat. Commun. 2016, 7, 11691 10.1038/ncomms11691. 27325212 PMC5512612

[ref16] Todescato F.; Capelli R.; Dinelli F.; Murgia M.; Camaioni N.; Yang M.; Bozio R.; Muccini M. Correlation between Dielectric/Organic Interface Properties and Key Electrical Parameters in PPV-Based OFETs. J. Phys. Chem. B 2008, 112, 10130–10136. 10.1021/jp8012255. 18661927

[ref17] Yang J.; Zhao Z.; Wang S.; Guo Y.; Liu Y. Insight into High-Performance Conjugated Polymers for Organic Field-Effect Transistors. Chem 2018, 4, 2748–2785. 10.1016/j.chempr.2018.08.005.

[ref18] Faure M. D. M.; Lessard B. H. Layer-by-Layer Fabrication of Organic Photovoltaic Devices: Material Selection and Processing Conditions. J. Mater. Chem. C 2021, 9, 14–40. 10.1039/d0tc04146g.

[ref19] Gon M.; Tanimura K.; Yaegashi M.; Tanaka K.; Chujo Y. PPV-Type π-Conjugated Polymers Based on Hypervalent Tin(IV)-Fused Azobenzene Complexes Showing near-Infrared Absorption and Emission. Polym. J. 2021, 53, 1241 10.1038/s41428-021-00506-x.

[ref20] Peters M.; Zaquen N.; D’Olieslaeger L.; Bové H.; Vanderzande D.; Hellings N.; Junkers T.; Ethirajan A. PPV-Based Conjugated Polymer Nanoparticles as a Versatile Bioimaging Probe: A Closer Look at the Inherent Optical Properties and Nanoparticle–Cell Interactions. Biomacromolecules 2016, 17, 2562–2571. 10.1021/acs.biomac.6b00574. 27345494

[ref21] Cheng F.; Zhang G.-W.; Lu X.-M.; Huang Y.-Q.; Chen Y.; Zhou Y.; Fan Q.-L.; Huang W. A Cationic Water-Soluble Poly(p-Phenylenevinylene) Derivative: Highly Sensitive Biosensor for Iron-Sulfur Protein Detection. Macromol. Rapid Commun. 2006, 27, 799–803. 10.1002/marc.200500867.

[ref22] Zaquen N.; Lu H.; Chang T.; Mamdooh R.; Lutsen L.; Vanderzande D.; Stenzel M.; Junkers T. Profluorescent PPV-Based Micellar System as a Versatile Probe for Bioimaging and Drug Delivery. Biomacromolecules 2016, 17, 4086–4094. 10.1021/acs.biomac.6b01653. 27936730

[ref23] Granier T.; Thomas E. L.; Gagnon D. R.; Karasz F. E.; Lenz R. W. Structure Investigation of Poly(p-Phenylene Vinylene). J. Polym. Sci., Part B: Polym. Phys. 1986, 24, 2793–2804. 10.1002/polb.1986.090241214.

[ref24] Zyung T.; Kim J.-J.; Hwang W.-Y.; Hwang D. H.; Shim H. K. Electroluminescence from Poly(p-Phenylenevinylene) with Monoalkoxy Substituent on the Aromatic Ring. Synth. Met. 1995, 71, 2167–2169. 10.1016/0379-6779(94)03205-k.

[ref25] Woo H. S.; Graham S. C.; Halliday D. A.; Bradley D. D. C.; Friend R. H.; Burn P. L.; Holmes A. B. Photoinduced Absorption and Photoluminescence in Poly(2,5-Dimethoxy-p-Phenylene Vinylene). Phys. Rev. B 1992, 46, 7379–7389. 10.1103/physrevb.46.7379. 10002473

[ref26] Thorn-Csányi E.; Kraxner P. All-Trans Oligomers of 2,5-Dialkyl-1,4-Phenylenevinylenes–metathesis Preparation and Characterization. Macromol. Chem. Phys. 1997, 198, 3827–3843. 10.1002/macp.1997.021981205.

[ref27] Carsten B.; He F.; Son H. J.; Xu T.; Yu L. Stille Polycondensation for Synthesis of Functional Materials. Chem. Rev. 2011, 111, 1493–1528. 10.1021/cr100320w. 21314190

[ref28] Bao Z.; Chan W. K.; Yu L. Exploration of the Stille Coupling Reaction for the Synthesis of Functional Polymers. J. Am. Chem. Soc. 1995, 117, 12426–12435. 10.1021/ja00155a007.

[ref29] Thorn-Csányi E.; Kraxner P. Synthesis of Soluble, All-Trans Poly(2,5-Diheptyl-p-Phenylenevinylene) via Metathesis Polycondensation. Macromol. Rapid Commun. 1995, 16, 147–153. 10.1002/marc.1995.030160209.

[ref30] Man P. C.; Crayston J. A.; Halim M.; Samuel I. D. W. Synthesis and Characterisation of Partially-Conjugated 2,5-Dialkoxy-p-Phenylenevinylene Lightemitting Polymers. Synth. Met. 1999, 102, 1081–1082. 10.1016/s0379-6779(98)01371-x.

[ref31] Chuah B. S.; Cacialli F.; dos Santos D. A.; Feeder N.; Davies J. E.; Moratti S. C.; Holmes A. B.; Friend R. H.; Brédas J. L. A Highly Luminescent Polymer for LEDs. Synth. Met. 1999, 102, 935–936. 10.1016/s0379-6779(98)00965-5.

[ref32] Klavetter F. L.; Grubbs R. H. Polycyclooctatetraene (Polyacetylene): Synthesis and Properties. J. Am. Chem. Soc. 1988, 110, 7807–7813. 10.1021/ja00231a036.

[ref33] Sutthasupa S.; Shiotsuki M.; Sanda F. Recent Advances in Ring-Opening Metathesis Polymerization, and Application to Synthesis of Functional Materials. Polym. J. 2010, 42, 905 10.1038/pj.2010.94.

[ref34] Song K.; Kim K.; Hong D.; Kim J.; Heo C. E.; Kim H. I.; Hong S. H. Highly Active Ruthenium Metathesis Catalysts Enabling Ring-Opening Metathesis Polymerization of Cyclopentadiene at Low Temperatures. Nat. Commun. 2019, 10, 3860 10.1038/s41467-019-11806-5. 31455772 PMC6712042

[ref35] Yu C.-Y.; Turner M. L. Soluble Poly(p-Phenylenevinylene)s through Ring-Opening Metathesis Polymerization. Angew. Chem., Int. Ed. 2006, 45, 7797–7800. 10.1002/anie.200602863. 17061303

[ref36] Lidster B. J.; Kumar D. R.; Spring A. M.; Yu C.-Y.; Turner M. L. Alkyl Substituted Poly(p-Phenylene Vinylene)s by Ring Opening Metathesis Polymerisation. Polym. Chem. 2016, 7, 5544–5551. 10.1039/c6py01186a.

[ref37] Komanduri V.; Tate D. J.; Marcial-Hernandez R.; Kumar D. R.; Turner M. L. Synthesis and ROMP of Benzothiadiazole Paracyclophane-1,9-Dienes to Donor–Acceptor Alternating Arylenevinylene Copolymers. Macromolecules 2019, 52, 7137–7144. 10.1021/acs.macromol.9b01244.

[ref38] Jia Z.; Qin S.; Meng L.; Ma Q.; Angunawela I.; Zhang J.; Li X.; He Y.; Lai W.; Li N.; Ade H.; Brabec C. J.; Li Y. High Performance Tandem Organic Solar Cells via a Strongly Infrared-Absorbing Narrow Bandgap Acceptor. Nat. Commun. 2021, 12, 178 10.1038/s41467-020-20431-6. 33420010 PMC7794321

[ref39] Scharber M. C.; Sariciftci N. S. Low Band Gap Conjugated Semiconducting Polymers. Adv. Mater. Technol. 2021, 6, 2000857 10.1002/admt.202000857.

[ref40] Bronstein H.; Nielsen C. B.; Schroeder B. C.; McCulloch I. The Role of Chemical Design in the Performance of Organic Semiconductors. Nat. Rev. Chem. 2020, 4, 66–77. 10.1038/s41570-019-0152-9. 37128048

[ref41] Mitchell R. H.; Otsubo T.; Boekelheide V. The Wittig Rearrangement of Some Thiacyclophanes. Tetrahedron Lett. 1975, 16, 219–222. 10.1016/s0040-4039(00)71827-2.

[ref42] Allen F. H.; Kennard O.; Watson D. G.; Brammer L.; Orpen A. G.; Taylor R. Tables of Bond Lengths Determined by X-Ray and Neutron Diffraction. Part 1. Bond Lengths in Organic Compounds. J. Chem. Soc., Perkin Trans. 2 1987, 12, S1–S19. 10.1039/p298700000s1.

[ref43] North M. ROMP of Norbornene Derivatives of Amino-Esters and Amino-Acids. Ring Opening Metathesis Polymerisation and Related Chemistry: State of the Art and Visions for the New Century; Khosravi E., Szymanska-Buzar T., Eds.; Springer Netherlands: Dordrecht, 2002; pp 157–166.

[ref44] Kumar D. R.; Lidster B. J.; Adams R. W.; Turner M. L. Mechanistic Investigation of the Ring Opening Metathesis Polymerisation of Alkoxy and Alkyl Substituted Paracyclophanedienes. Polym. Chem. 2017, 8, 3186–3194. 10.1039/c7py00543a.

[ref45] Yu C.-Y.; Horie M.; Spring A. M.; Tremel K.; Turner M. L. Homopolymers and Block Copolymers of P-Phenylenevinylene-2,5-Diethylhexyloxy-p-Phenylenevinylene and m-Phenylenevinylene-2,5-Diethylhexyloxy-p-Phenylenevinylene by Ring-Opening Metathesis Polymerization. Macromolecules 2010, 43, 222–232. 10.1021/ma901966g.

[ref46] Wang F.; He F.; Xie Z. Q.; Li Y. P.; Hanif M.; Li M.; Ma Y. Poly(p-Phenylene Vinylene) Derivatives with Different Contents of Cis-Olefins and Their Effect on the Optical Properties. Macromol. Chem. Phys. 2008, 209, 1381–1388. 10.1002/macp.200800047.

