# Supplementary material for: Synthesis and Ring-Opening Metathesis Polymerization of o-Dialkoxy Paracyclophanedienes
Source: Macromolecules. 2022 Dec 7;55(24):10854–64. doi: 10.1021/acs.macromol.2c02111 (PMC9798985; doi:10.1021/acs.macromol.2c02111)
Supplement: Supplementary file 1 — ma2c02111_si_001.pdf [file ma2c02111_si_001.pdf]

Supporting Information

Synthesis and Ring Opening Metathesis Polymerisation of *o*-Dialkoxy Paracyclophanedienes

Yurachat Janpatompong, Andrew M. Spring, Venukrishnan Komanduri, Raja U. Khan and Michael L. Turner*

Department of Chemistry, University of Manchester, Oxford Road, M13 9PL, United Kingdom

Table of Contents

S1. General experimental details for synthesis 1

S2. Computational details 2

S3. Synthesis of paracyclophanediene monomers M1 2

S4. ROMP of monomers M1 with G2 catalyst; *in-situ* ^{1}H NMR experiments 14

S5. ROMP of monomers M1 with G2 catalyst 16

S6. ROMP of monomers M1 with G3 catalyst-(Microwave) 16

S7. Sequential ROMP of monomers M1 and M2 with G2 catalyst-synthesis of fully conjugated donor-acceptor diblock copolymer 16

S8. *Cis-trans* isomerisation 17

S9. Matrix-assisted laser desorption/ionisation time-of-flight mass spectrometry (MALDI-TOF-MS) of polymer 8a 28

S10. Molecular weight data and SEC traces 29

S11. Optical properties of polymers 31

S12. Electrochemical properties of polymers 33

S13. References 36

S1. General experimental details for synthesis

Nuclear magnetic resonance (NMR) spectra were recorded in deuterated chloroform and dichloromethane with chemical shift obtained using residual non deuterated solvent as an internal standard (^{1}H NMR spectroscopy; 7.23 ppm for chloroform-*d* and 5.23 ppm for dichloromethane-d_2) on Bruker spectrometers operating at either 400 MHz or 500 MHz. The following abbreviations are used to indicate the multiplicity of the signals; s = singlet, d = doublet, m = multiplet, br(m) = broad multiplet. Matrix-assisted laser desorption/ionization time-of-flight mass spectrometry (MALDI-TOF-MS) were carried out on a Shimadzu Biotech AXIMA Confidence MALDI mass spectrometer in linear (positive) mode. Calibration was conducted against poly(propylene glycol) (M_n = 4.0 kg mol^{-1}) or Polymer Factory SpheriCal® MALDI-TOF-MS calibration standards (series of four monodisperse dendrimers in mass range 1716.82-3424.63 Da) were indicated. The polymer solution 50 µL (1 mg mL^{-1} in THF) was mixed with 50 µL of a 10 mg mL^{-1} solution of the matrix (dithranol) in THF. A drop of this solution was spotted onto a MALDI plate which had been pre-spotted with sodium iodide in THF (10 mg mL^{-1}).

Atmospheric pressure chemical ionization (APCI) and high-resolution electrospray mass spectrometry (HRMS) measurements for all the synthetic intermediates were carried out using Agilent 6120 Quadrapole LC/MS and Thermo Scientific Exactive Plus EMR (extended mass range) Orbit respectively. Gel permeation chromatography (GPC) analyses were performed in THF solution (~1 mg mL^{-1}) at 40 °C using a GPC Agilent 1260 Infinity II with 2 × PL gel 10 µm mixed-B and a PL gel 500 Å column, and equipped with a differential refractive index (DRI) detector employing narrow polydispersity polystyrene standards (Agilent Technologies) as a calibration reference. Samples were filtered through a Whatman Puradisc 4 mm syringe filter with 0.45 µm PTFE membrane before injection to equipment, and experiments were carried out with injection volume of 50 µL, flow rate of 1 mL min^{-1}. Results were analyzed using Agilent GPC/SEC Software Version 2.2. The analysed samples contained *n*-dodecane as a flow marker. UV-Vis absorption spectra and optical densities were recorded on a Varian Cary 5000 UV-Vis-NIR spectrophotometer and Fluorescence emission spectra were obtained on a Varian Cary Eclipse fluorescence spectrophotometer. Fluorescence quantum yields for all the polymers were measured in dilute chloroform solutions on HAMAMATSU Absolute PL Quantum Yield Spectrometer C11347 using integration sphere method. Slow addition was performed using a 205S Watsons-Marlow peristaltic pump. The electrochemical CV were performed on a computer controlled CompactStat.h (Ivium Technologies ©) in a three-electrode configuration with a glassy carbon disk, Pt wire and Ag/Ag+ electrode as the working electrode, counter electrode, and reference electrode, respectively in a 0.1 M tetrabutylammonium hexafluorophosphate (NBu_4PF_6) acetonitrile solution as supporting electrolyte, at a scan rate of 50mV s^{-1} . The polymers solutions were drop casted on the glassy carbon disk and left to dry at room temperature before conducting the measurements. The potential of Ag/Ag+ reference electrode was internally calibrated by using Ferrocene/Ferrocenium (Fc/Fc+) redox couple.

Unless otherwise noted, all reagents were used as received from Sigma-Aldrich and Lancaster without further purification. Anhydrous THF used for all the polymerization reactions was purchased from Sigma-Aldrich and degassed by freeze-pump-thaw technique (three times). All other anhydrous solvents and reagents were purchased from Sigma-Aldrich, Fisher Scientific, Alfa Aesar or Acros and used as received. Reactions were monitored by thin layer chromatography (TLC) carried out on DC-Fertigfolie POLYGRAM® SIL G/UV_{254}, using UV light as the visualizing agent. Flash silica gel chromatography was performed using SiliCycle SiliaFlash® Irregular Silica Gel (60 Å, 230−400 mesh).

S2. Computational details

All calculations were performed by the Gaussian09 program package and GaussView 5.0.8 molecular visualization program. The molecular structures were created and optimized using the Density Functional Theory (DFT)[1], single point calculations with the B3LYP (Becke three-parameter hybrid correlation functional combined with Lee–Yang–Parr correlation functional)[2], with 6-311G(d,p) basis set. Ring strain is calculated as an energy difference between optimized ring-closed **M1** with an H_2 molecule (for atom balance) and the ring-opened monomeric form featuring a *cis-trans* microstructure with H-atoms inserted at the position of ring-opening. The band gap values ($E_g = E_{HOMO}-E_{LUMO}$) of the oligomers were calculated using B3LYP/6-311G(d,p) level to obtain the highest occupied molecular orbital (HOMO) and lowest unoccupied molecular orbital energies (LUMO).

S3. Synthesis of paracyclophanediene monomers M1

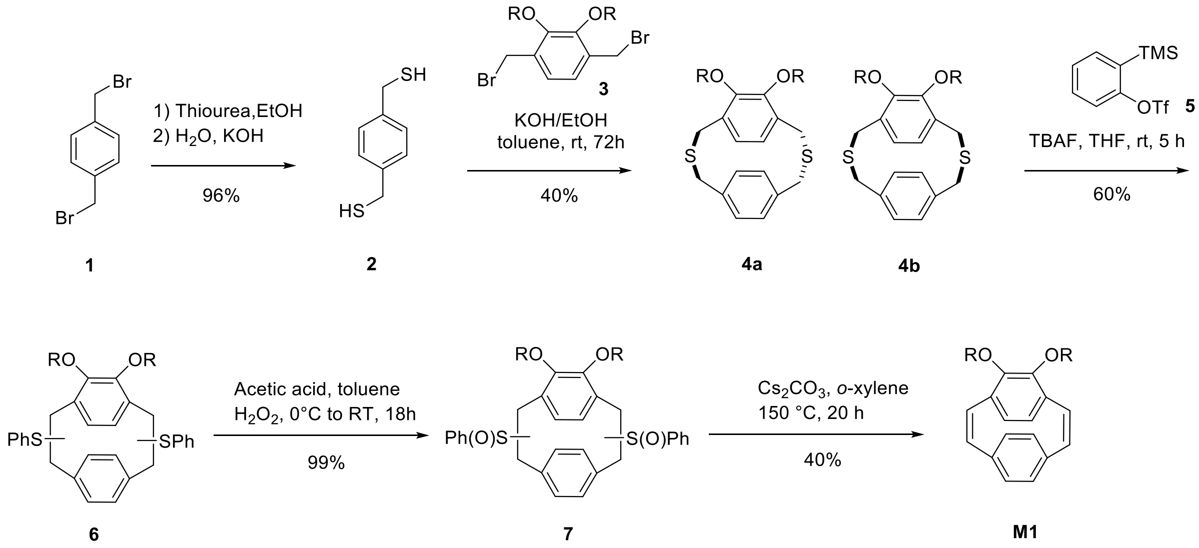

Synthesis of 6,7-Diethylhexyloxy-2,11-dithia[3.3]paracyclophane conformers (4a,b)

Compound **2** (6.54 g, 38.43 mmol) and compound **3** (20 g, 38.43 mmol) were dissolved in deoxygenated toluene (1000 mL). This solution was added dropwise to KOH (5.92 g, 105.46 mmol) in deoxygenated ethanol (2200 mL) over 72 hours followed by stirring for an additional 6 hours at

room temperature. The solvent was removed *in vacuo* and the residue was dissolved in dichloromethane (500 mL). The organic layer was washed with water (2x500 mL), brine (200 mL) followed by drying (anhydrous $MgSO_4$). The resulting yellow oil was dissolved in a solvent system of 10% DCM and 90% petroleum ether and purified by column chromatography. Collection of the two main fractions gave 6,7- diethylhexyloxy-2,11-dithia[3.3]paracyclophane conformers **(4a,b)** in a yield of 40% combined (8.03g). **^{1}H-NMR ($CDCl_3$, 500MHz): 4a** δ 6.94 (s, 2H), 6.81 (s, 2H), 6.41 (s, 2H), 4.02 (d, J = 15Hz, 2H), 3.99 (2x2dd, J_1 = 9Hz, J_2 = 6Hz, 2H), 3.90 (d(AB), J = 15 Hz, 2H), 3.68-3.76 (2x2dd, J_1 = 9Hz, J_2 = 6Hz, 2H), 3.75 (d(AB), J = 15Hz, 2H), 3.46 (d, J = 15 Hz, 2H), 1.82 (sep, J= 6 Hz, 2H), 1.23-1.75 (m, 16H), 0.84-1.03 (4xt, J = 7 Hz, 12H) ppm. **^{13}C-NMR** (**$CDCl_3$, 500MHz**): δ 148.85, 148.83, 135.89, 129.75, 129.73, 129.05, 129.03, 128.89, 126.20, 126.17, 75.95, 75.93, 75.90, 75.87, 40.57, 40.44, 38.23, 32.78, 32.75, 30.49, 30.22, 30.21, 29.24, 29.12, 29.10, 23.78, 23.66, 23.19, 23.15, 14.15, 14.14, 14.12, 11.32, 11.09 ppm. **MS(HRMS):** 528.3214 $[M+H]^+$ (calcd. for $C_{32}H_{48}O_2N_2S_2$+H: 528.3090). **4b** δ 7.01 (s, 4H), 6.54 (s, 2H), 3.71-3.79 (4xdd, 4H), 3.60 (s, 4H), 3.56 (s, 4H), 1.76 (sep, J = 6 Hz, 1H), 1.62-1.51 (sep, J = 6 Hz, 1H), 1.51-1.25 (16H), 0.94 (2xt, J = 7 Hz, 12H) ppm. **^{13}C-NMR** (**$CDCl_3$, 500MHz**): 150.70, 137.12, 131.74, 128.74, 124.60, 76.27, 40.45, 36.13, 30.68, 30.30, 29.16, 23.64, 23.15, 14.13, 11.20, 11.18 ppm. **MS(HRMS):** 528.0124 $[M+H]^+$ (calcd. for $C_{32}H_{48}O_2N_2S_2$+H: 528.3090)

Synthesis of (51E,53E)-12,13-bis((2-ethylhexyl)oxy)-3,7-diphenyl-3,7-dithia-1(1,4)-benzena-5(1,4)-cyclohexanacyclooctaphane-51,53-diene 3,7-dioxide (6)

6,7-Diethylhexyloxy-2,11-dithia[3.3]paracyclophane conformers **(4a,b)** (5.00 g, 9.45 mmol) and 2-(trimethylsilyl)phenyl trifluoromethanesulfonate (7.05 g, 23.64 mmol) were dissolved in anhydrous THF (50 mL) and stirred at room temperature for 5 min. To this solution, TBAF·3H_2O (8.95 g, 28.30 mmol) dissolved in anhydrous THF (30 mL) was added dropwise using a syringe pump over a period of 5 hours. The resulting solution was stirred for additional one hour and the solvent removed *in vacuo* revealing a brown oil. The crude was purified by silica gel flash column chromatography (gradient: 0-30% dichloromethane/petroleum ether) to obtain the desired product **6** (mixture of diastereomers , 4.00 g, 60 % yield) as a clear oil. **Mass spectrum (APCI+, *m/z*)** 681 (M^+). **Mass spectrum (EI+, *m/z*)** 681 (M+).

Synthesis of 6,7-diethylhexyloxy-[2.2]paracyclophane-1,9-diene (M1)

Hydrogen peroxide (0.41ml, 4.02mmol, 0.136g, 30 wt % in acetic acid) was added dropwise over a period of 30 minutes to a solution of bisphenylsulphide isomers **(6)** (1.37 g, 2.01mmol) in toluene (60 ml) and acetic acid (20 ml) at 0 ºC under argon atmosphere. The vessel was allowed to warm up to room temperature and stirred for an additional 12 hours. The organic layer was washed with water (50 mL) and brine (50 mL). The organic layer was dried over magnesium sulfate, filtered and the solvent removed *in vacuo*. A clear viscous oil **7** (1.42 g) (99%) was obtained and used in the next step without any further purification. **Mass spectrum (APCI+, *m/z*)** 713 (M^+).

A solution of the mixture of *bis*-sulfoxides **7** (1.42 g, 1.99 mmol) was dissolved in deoxygenated anhydrous *o*-xylene (70 mL) and cesium carbonate (2.59 g, 7.94 mmol) was added and the suspension was heated under reflux for 20 hours. Upon consumption of the starting material (TLC) the reaction was cooled to room temperature, filtered and the solvent removed *in vacuo* to reveal a dark brown oil. The crude mixture was dissolved in ethyl acetate (100 mL), washed with water (50 mL), brine (50 mL) followed by drying (anhydrous $MgSO_4$). This oil was then dissolved in a solvent system of 40% DCM and 60% hexane and purified *via* column chromatography. Collection of the main fraction gave the pure product **M1** as yellowish oil in 40% yield (370 mg). **Mass spectrum (APCI+, *m/z*)** :461 $[M+H]^+$ **^{1}H-NMR (500MHz, $CDCl_3$):** δ 7.08 (d(AB), J = 10 Hz, 2H), 6.88 (d(AB), J = 10Hz, 2H), 6.87 (s, 2H), 6.60 (s, 2H), 6.05 (s, 2H), 4.00-3.87 (2xdd, J_1 = 9Hz, J_2 = 6Hz, 2H), 3.73-3.63 (2xdd, J_1 = 9Hz, J_2 = 6Hz, 2H), 1.68 (m, 2H), 1.29-1.60 (m, 16H), 0.95 (4xt, J = 7Hz, 12H) ppm. **^{13}C-NMR (500MHz, $CDCl_3$):** δ 151.75, 151.65, 151.56, 138.02, 135.56,133.62, 133.57, 129.92, 129.81, 129.85, 129.53, 129.78, 129.67, 128.35, 126.24, 126.12,126.29, 126.27, 75.78, 75.74, 75.53, 75.62, 40.39, 40.38, 30.45, 30.23, 29.26, 29.02,23.75, 23.63, 23.15, 23.12, 14.15, 14.12, 11.24, 10.98 ppm.

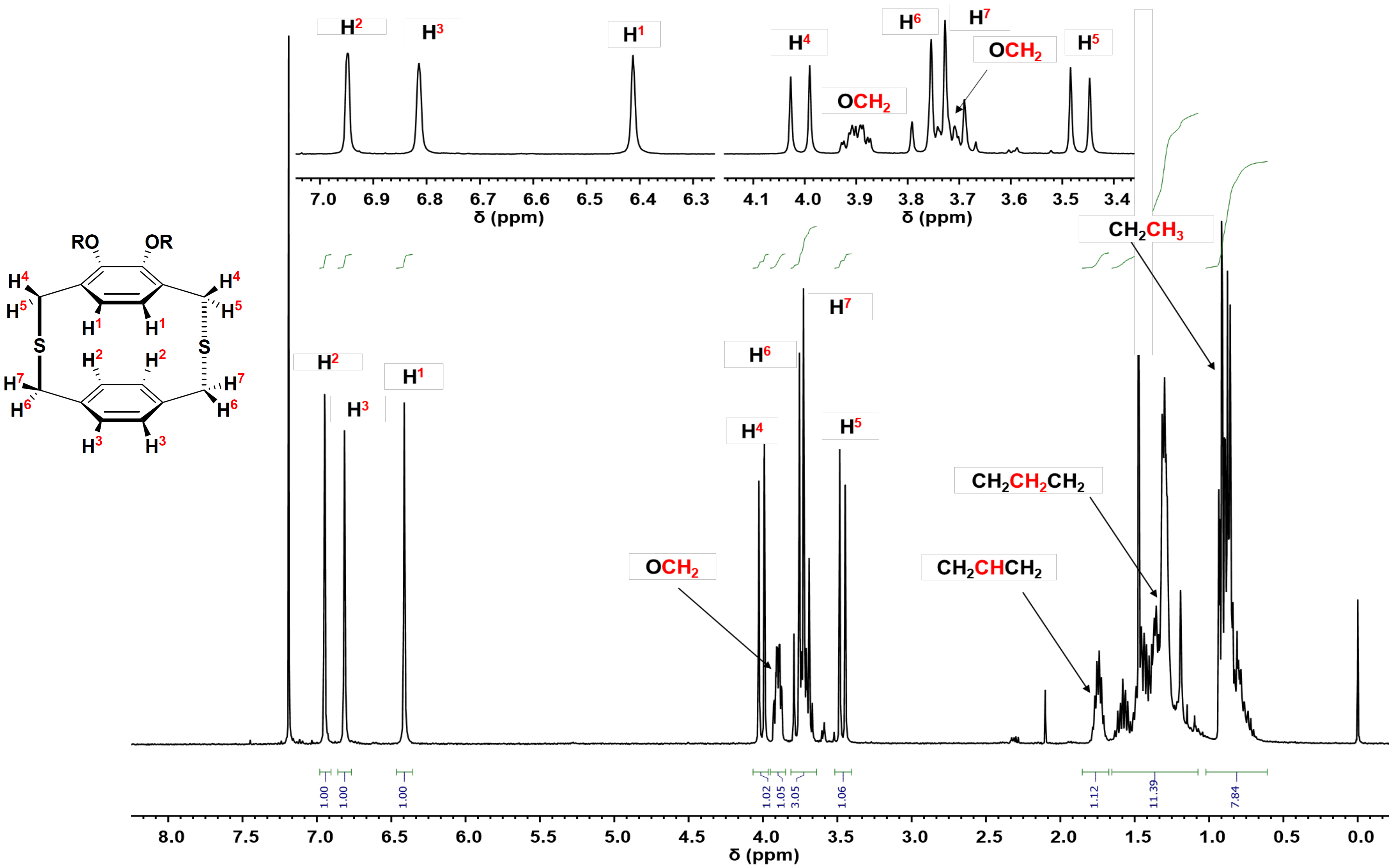

Figure S1 ^{1}H NMR spectrum of intermediate **4a** in $CDCl_3$.

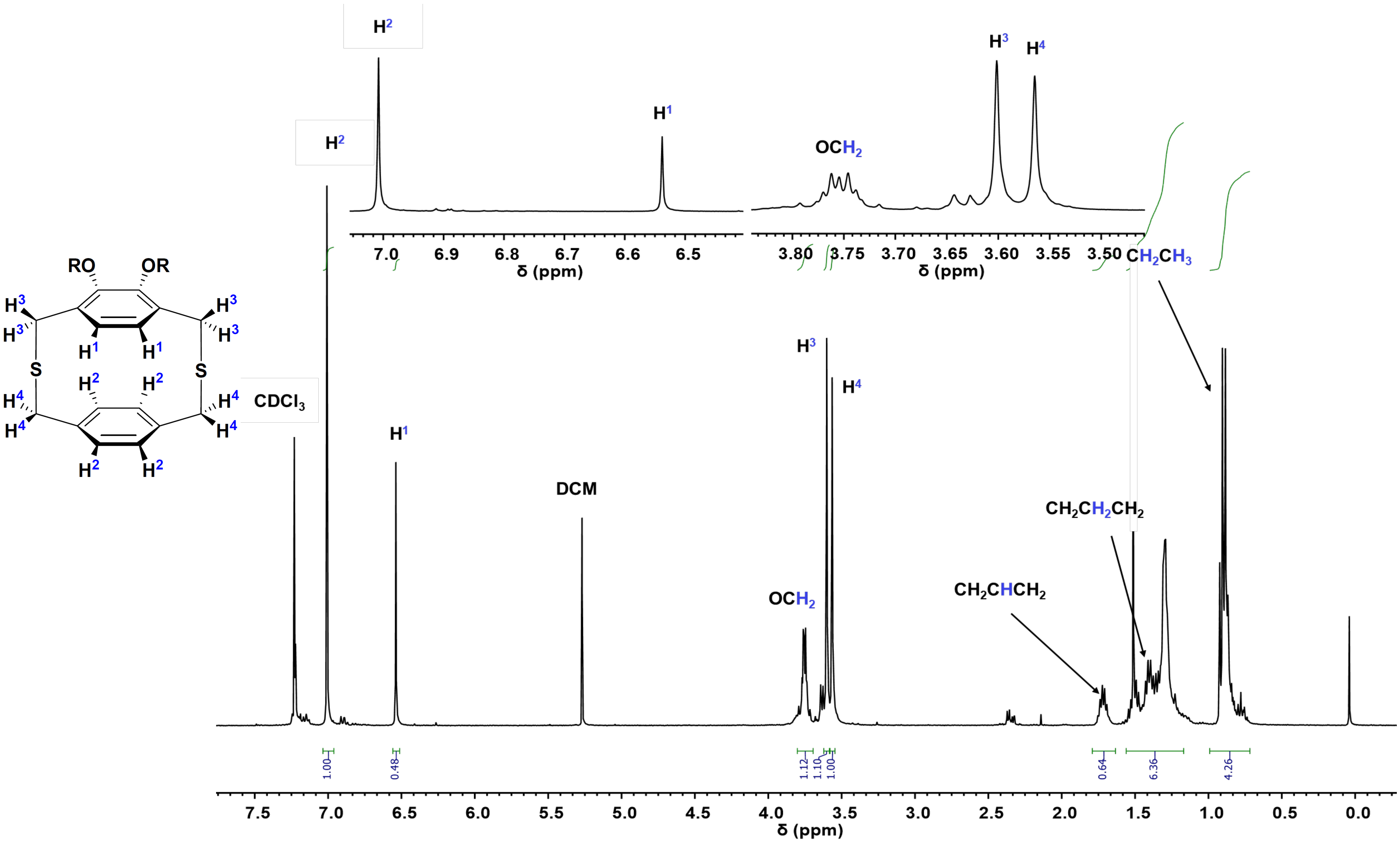

Figure S2 ^{1}H NMR spectrum of intermediate **4b** in $CDCl_3$.

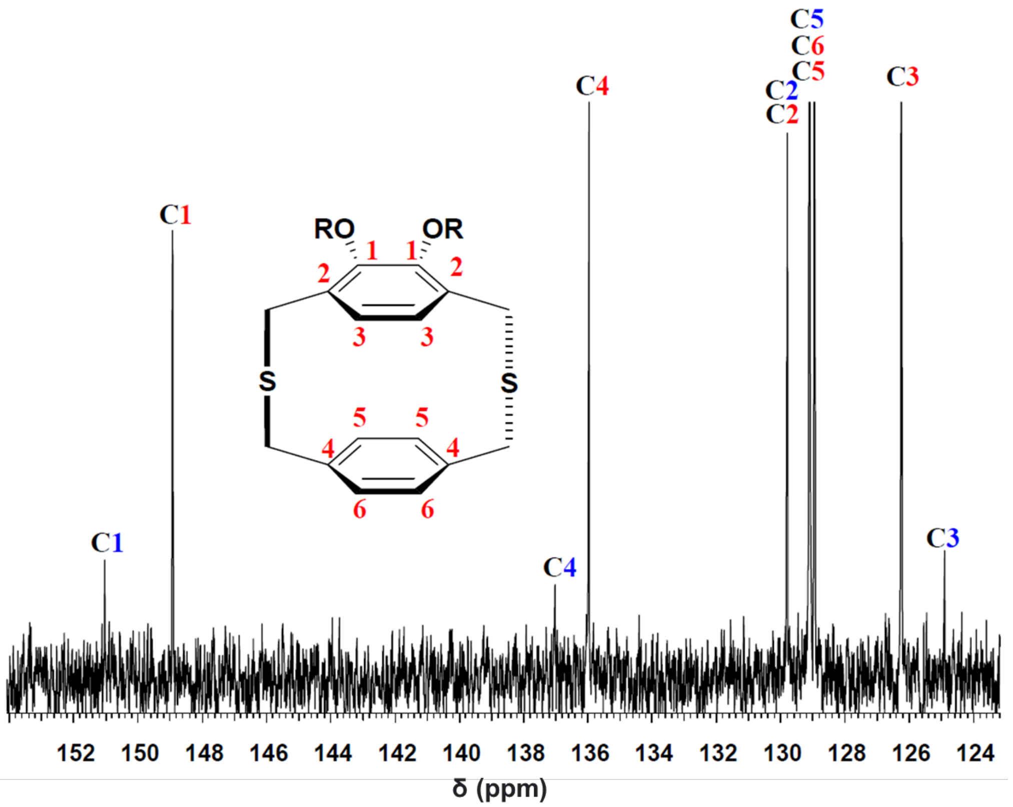

Figure S3 ^{13}C NMR spectrum of intermediate **4a** and **4b** in $CDCl_3$.

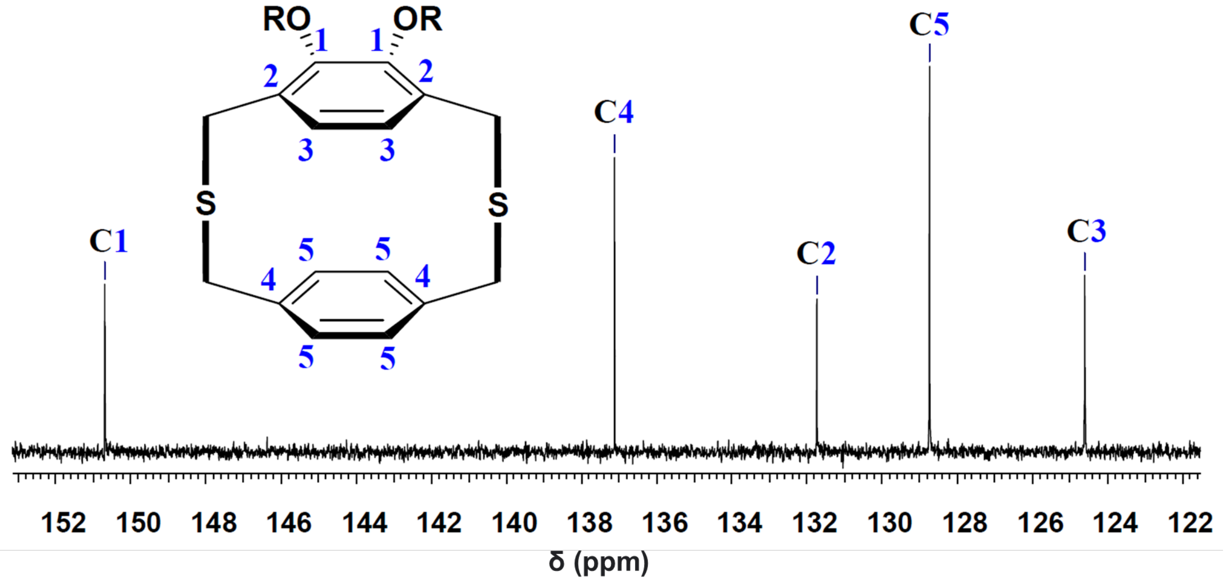

Figure S4 ^{13}C NMR spectrum of intermediate **4b** in $CDCl_3$.

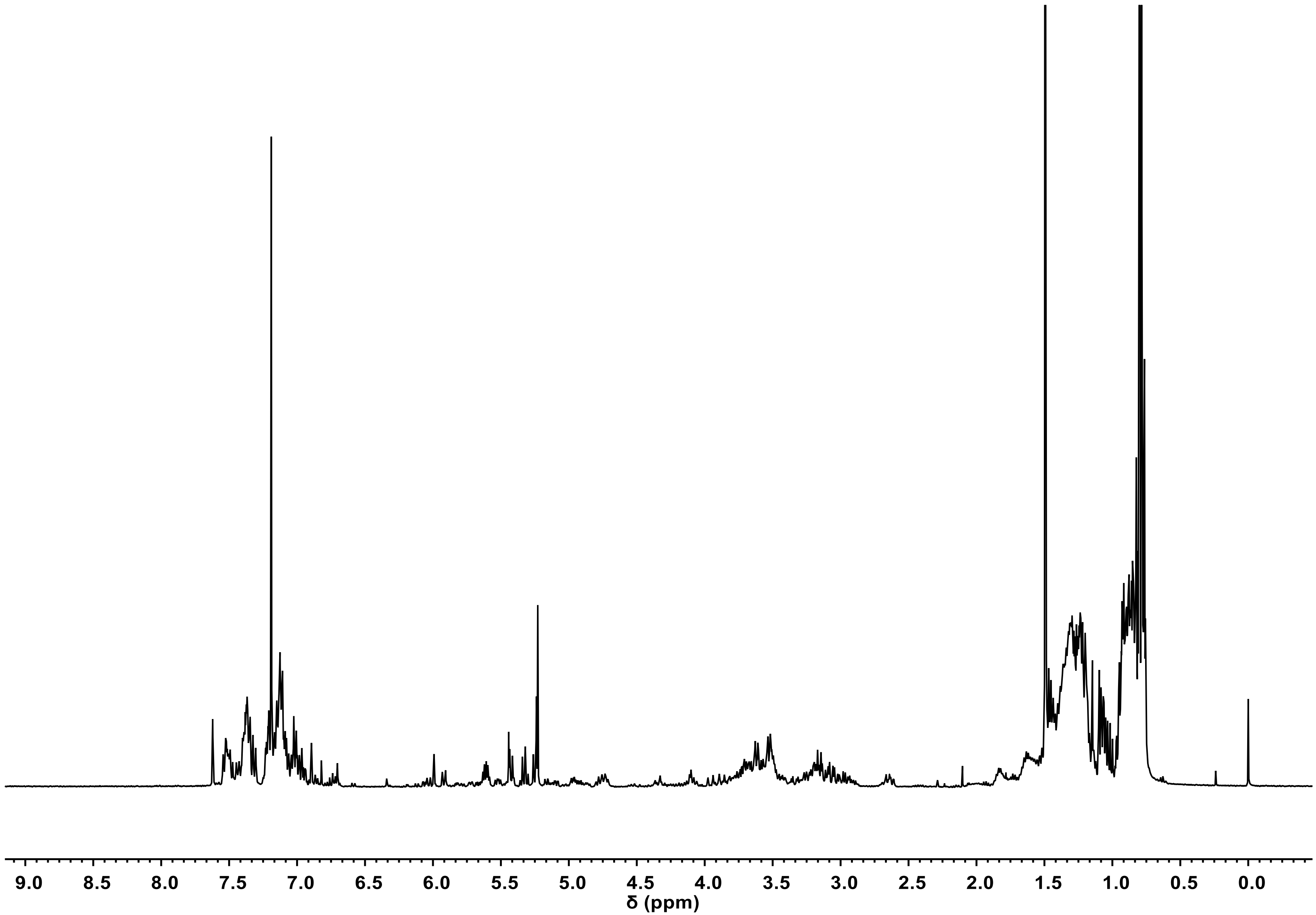

Figure S5 ^{1}H NMR spectrum of intermediate **7** in $CDCl_3$.

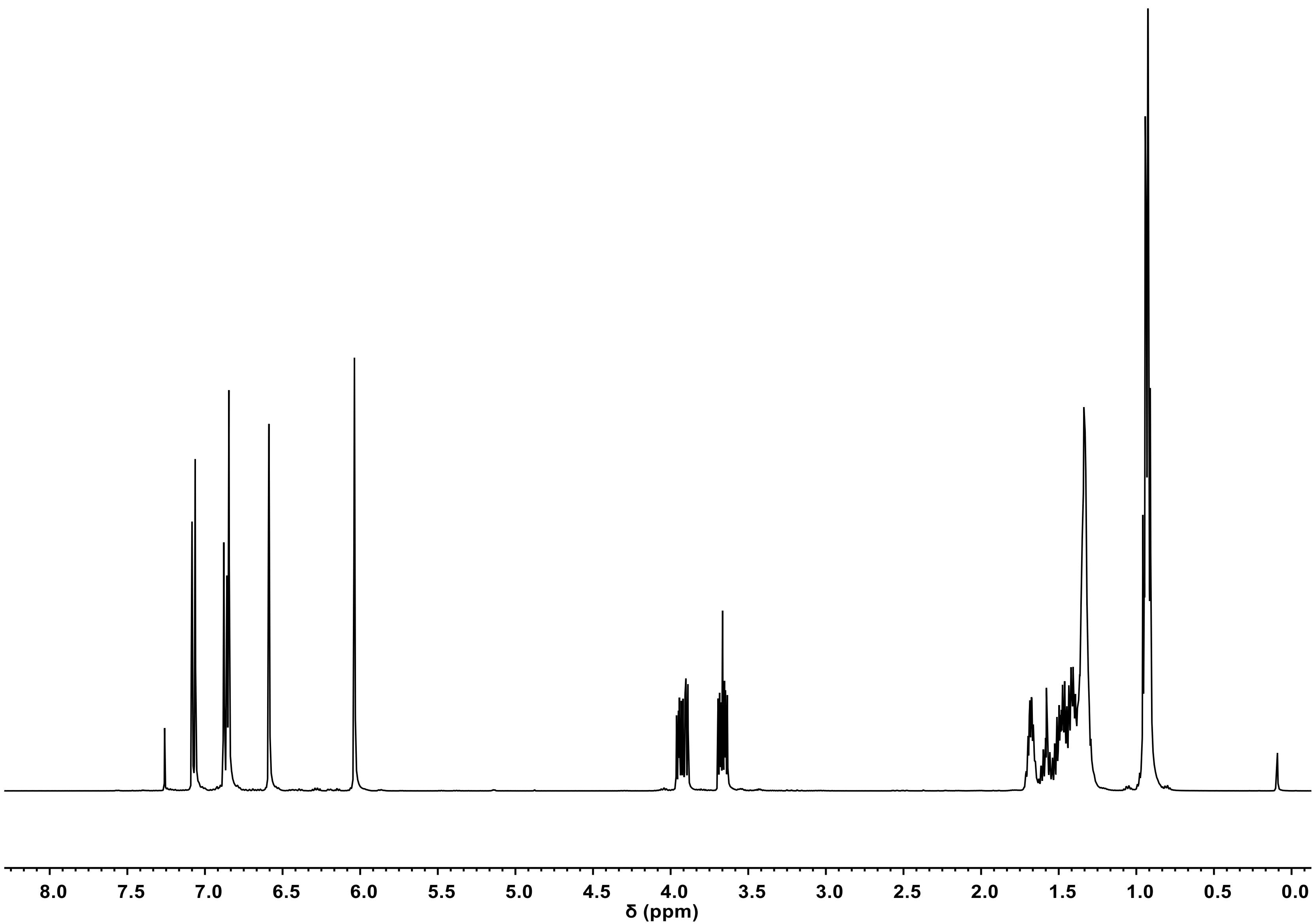

Figure S6 ^{1}H NMR spectrum of monomer **M1** in $CDCl_3$.

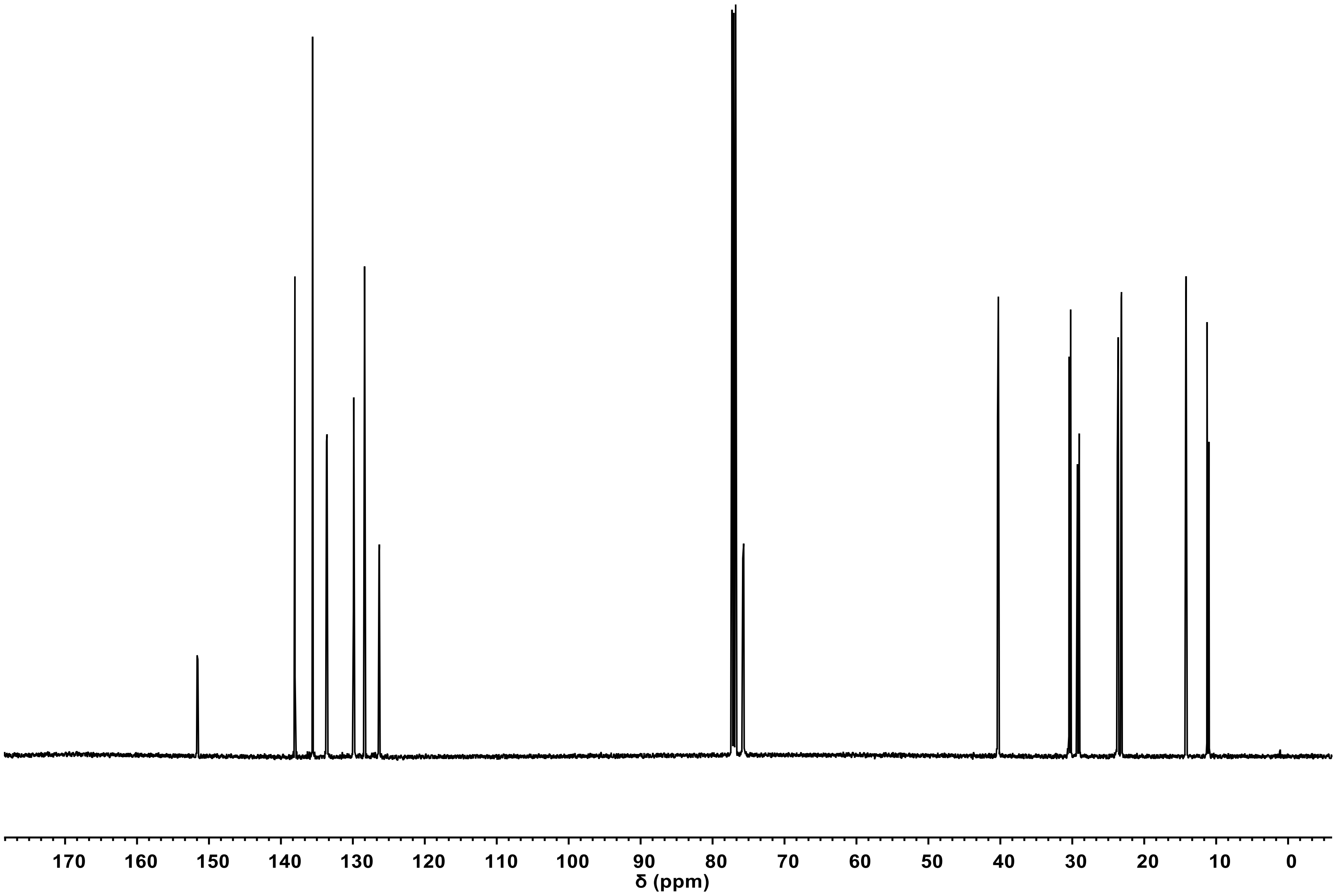

Figure S7 ^{13}C NMR spectrum of monomer **M1** in $CDCl_3$.

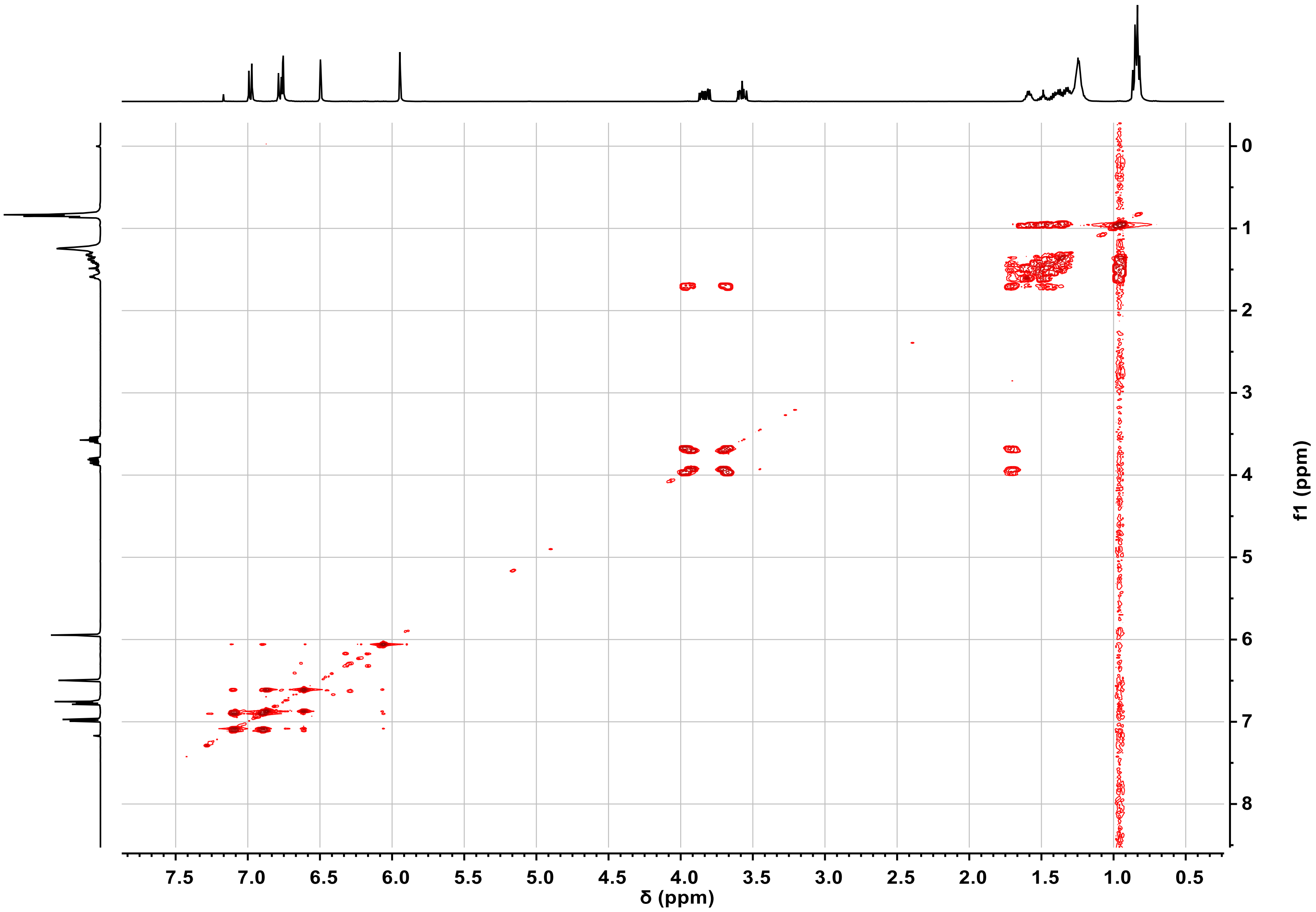

Figure S8 ^{1}H-^{1}H COSY NMR spectrum of monomer **M1** in $CDCl_3$.

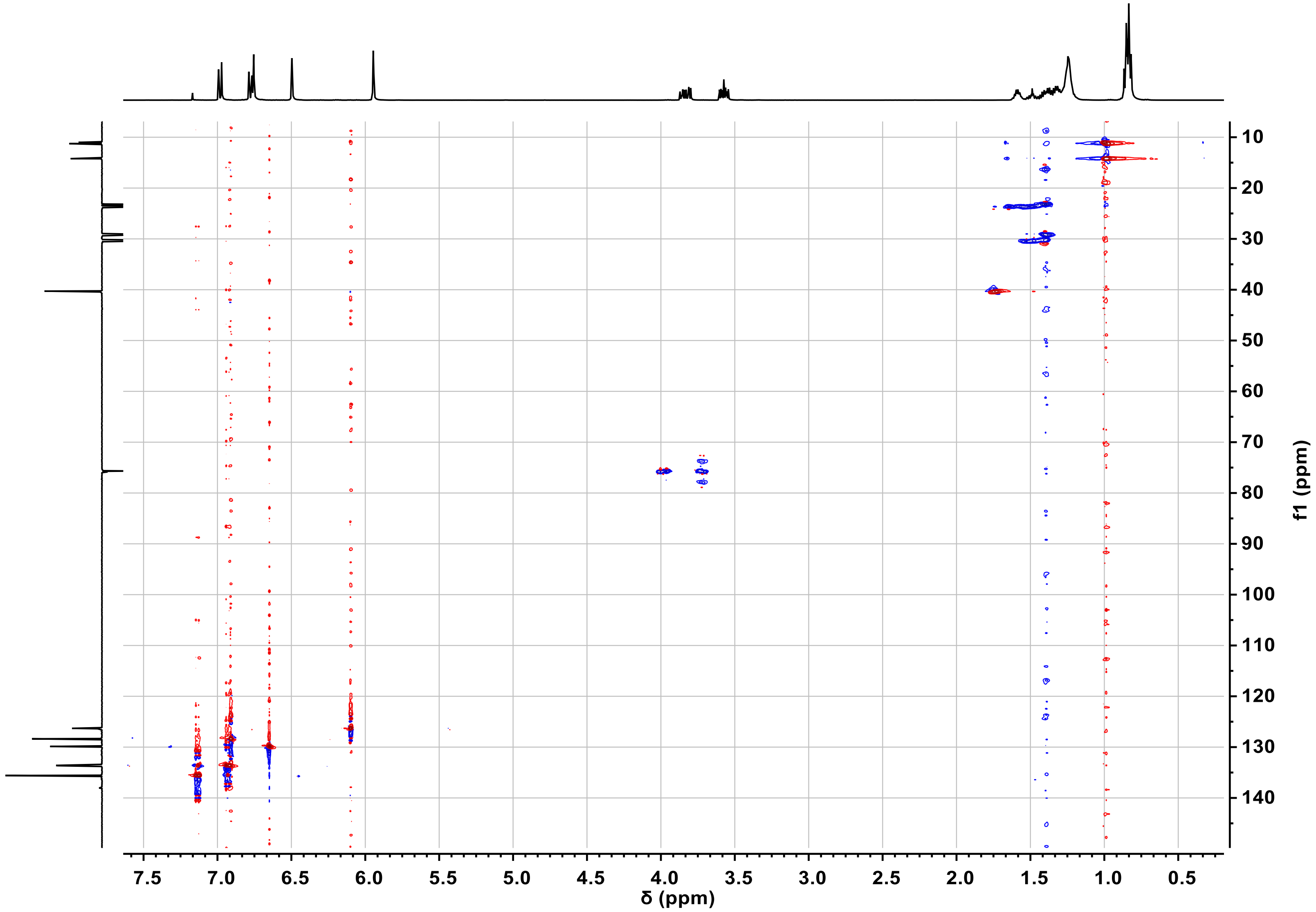

Figure S9 HSQC spectrum of monomer **M1** in $CDCl_3$.

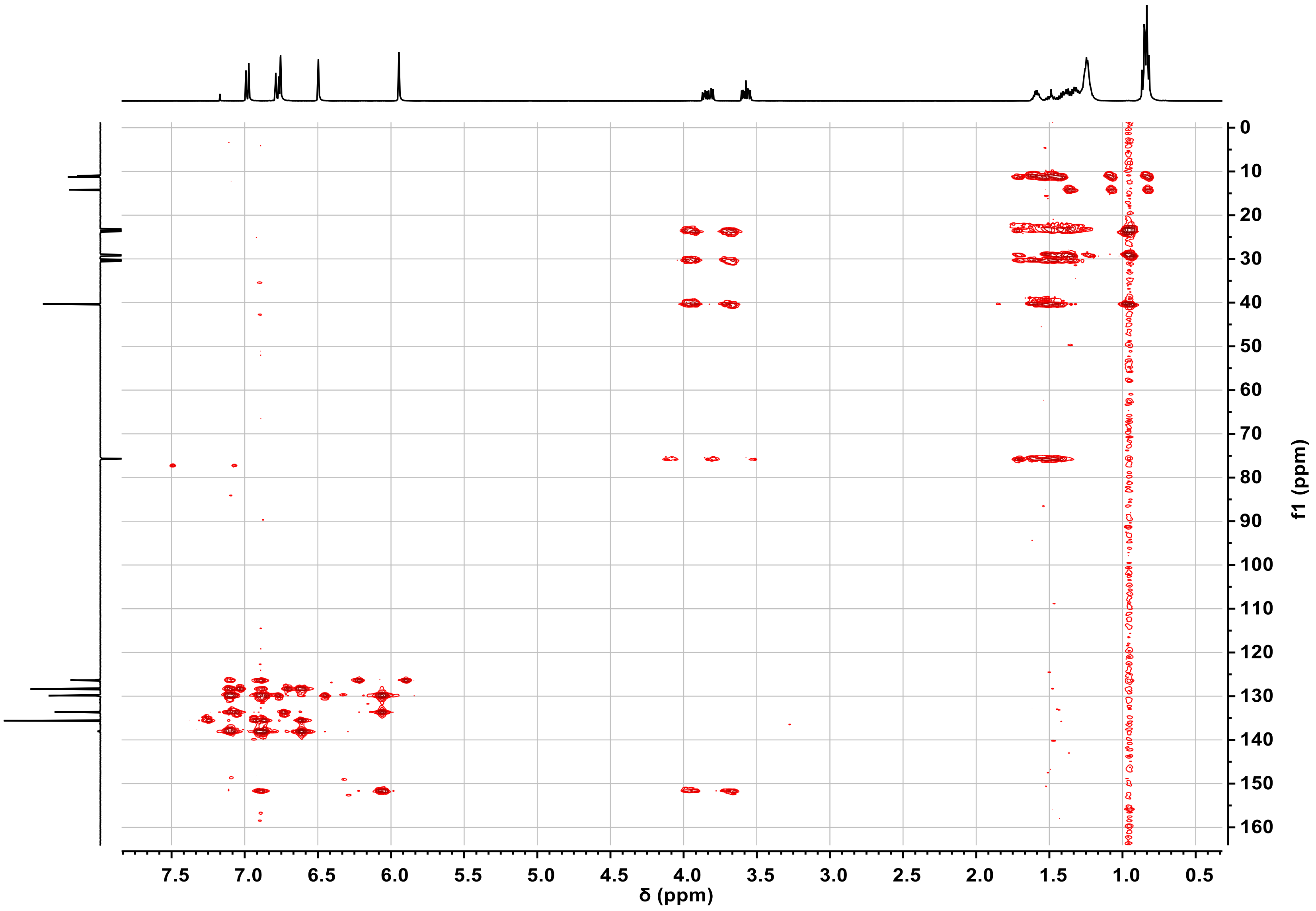

Figure S10 HMBC spectrum of monomer **M1** in $CDCl_3$.

S4. ROMP of monomers M1 with G2 catalyst; *in-situ* ^{1}H NMR experiments

General experimental procedure: **G2** (5.53mg, 6.51µmol catalyst and **M1** (30 mg, 65.12 µmol) were added into separate vials and transferred into an argon filled glovebox. **G2** was dissolved in THF-d_8 ([M] = 100 mM) and this catalyst solution was added to the vial containing monomer **M1** and mixed until homogeneous. The solution was transferred into a Young's NMR tube, sealed, removed from the glovebox, and kept in an ice bath containing NaCl. The NMR spectrometer was then set to the desired temperature at 25 or 55 °C, and NMR spectra were recorded at 5 min intervals throughout the polymerization. At the end of the reaction mixture was quenched with excess of deoxygenated ethyl vinyl ether in a vial and stirred at room temperature for 4 h . The reaction was precipitated into a short methanol/celite column followed by extraction of the polymer with chloroform. After evaporation of chloroform, polymer was isolated as yellow amorphous films.

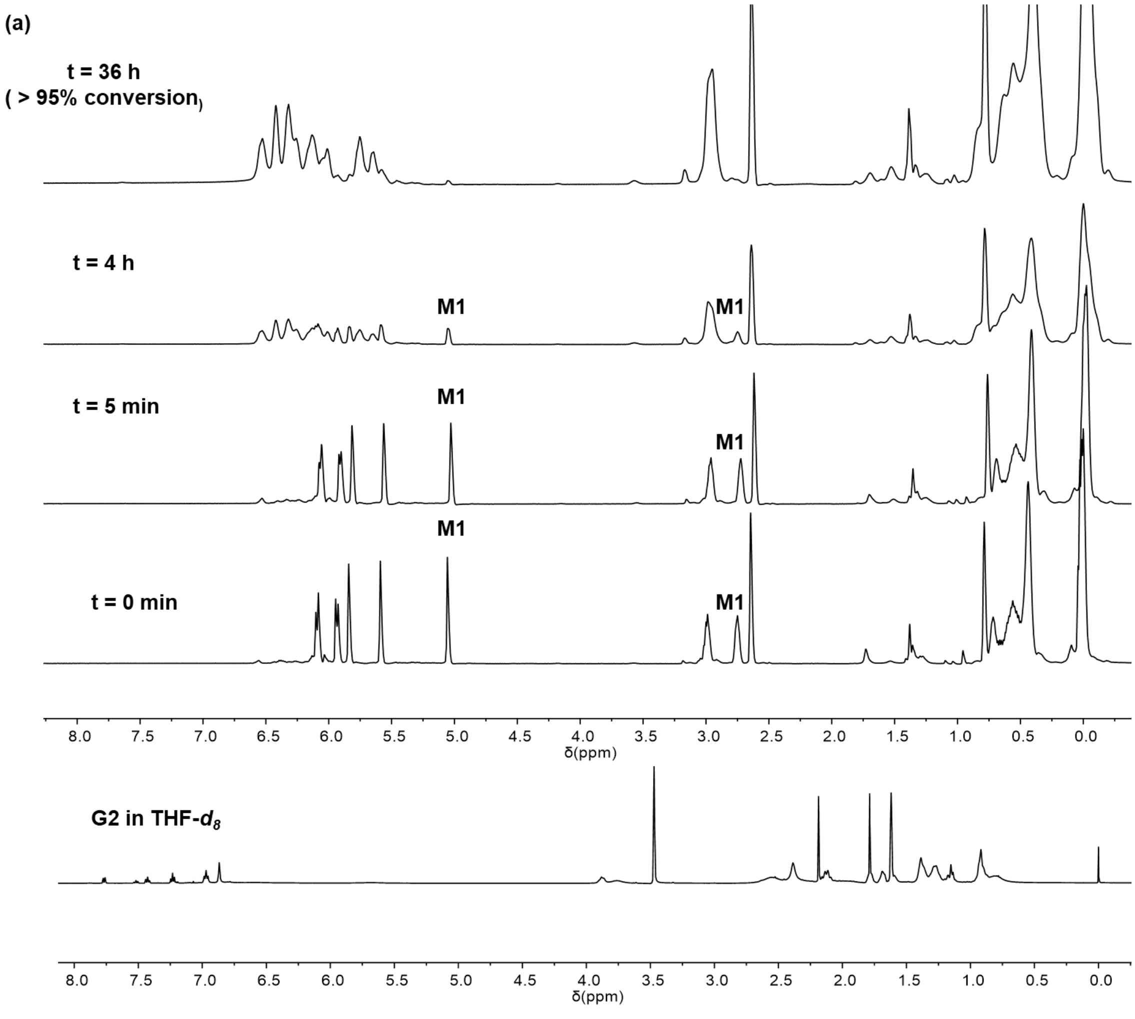

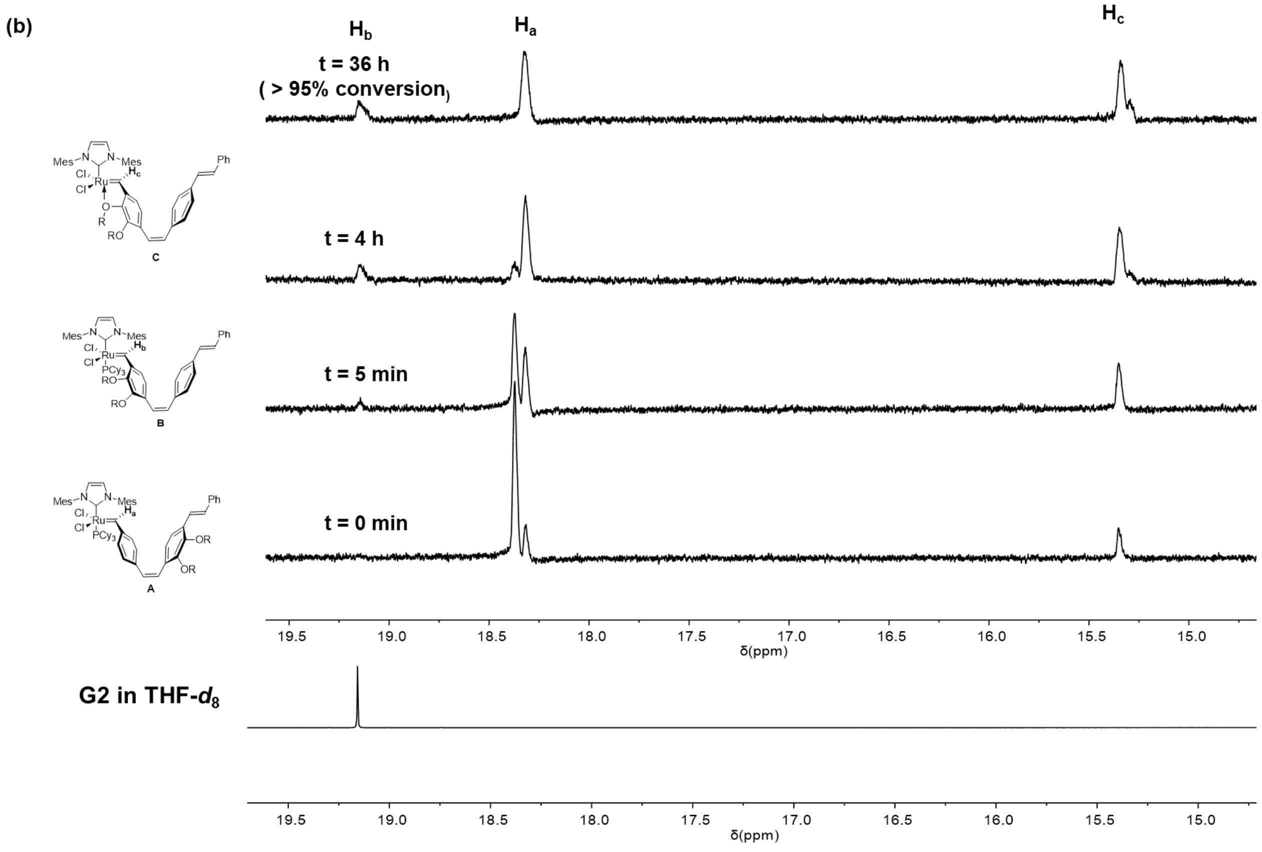

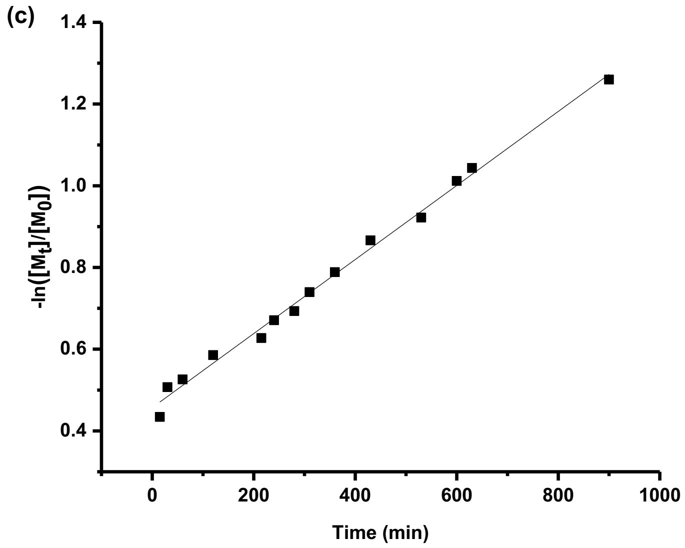

Figure S11 ROMP of monomer **M1** with **G2** catalyst; (a) *in-situ*^{1}H NMR experiment in THF-d_8 at 55 ^{o}C (b) ^{1}H NMR stack of monomer consumption carbene region (c) ROMP kinetics for propagation of **G2** at 55 °C.

S5. ROMP of monomers M1 with G2 catalyst

In an argon filled glovebox, a solution of **G2** (5.53 mg, 6.51μmol) in anhydrous, degassed THF ([**M1**] = 100 mM) was added into a vial containing cyclophanediene monomer **M1** (30 mg, 65.12 μmol). The vial was sealed, wrapped in foil and mixed at room temperature for 10 minutes. The reaction was placed in a preheated oil bath at 60 °C and stirred until complete monomer conversion observed by SEC and TLC. The reaction was cooled to room temperature and deoxygenated ethyl vinyl ether was added to quench the reaction. After stirring for a further 4 h at room temperature, the crude reaction mixture was purified *via* precipitation into methanol/Celite plug followed by extraction of the polymer with chloroform. The chloroform layer was evaporated under reduced pressure to give polymers as yellow amorphous films.

S6. ROMP of monomers M1 with G3 catalyst-(Microwave)

An argon filled CEM microwave reactor tube was charged with (20mg/0.0434mmol) of 6,7-diethylhexyloxy-[2.2]paracyclophane-1,9-diene **(M1)** Grubbs 3rd generation catalyst (3.84mg/4.34x10^{-3}mmol (n=10) was placed in an argon filled round bottom flask, this was then dissolved in anhydrous (2 ml) 1,2-dichloroethane. This solution was then injected by syringe into the microwave tubes. The tube was then agitated thoroughly to ensure a homogeneous mixture. The microwave tube was then transferred to the CEM microwave reactor and heated at a temperature of 80 ºC for a period of 30 minutes. An excess (2ml) of argon degassed ethyl vinyl ether was then injected into the microwave tube to terminate the polymerisation. A small magnetic stirrer bar was added to the tubes and the mixtures were stirred overnight at room temperature using a magnetic stirrer. After this, the polymer was precipitated into a methanol filled celite plug under *vacuo* and washed with an excess of methanol. The polymer was then redissolved in dichloromethane, removed from the celite and the evaporation of the solvent *in vacuo* allowed the isolation of the polymer **8d** as a glassy solid.

S7. Sequential ROMP of monomers M1 and M2 with G2 catalyst-synthesis of fully conjugated donor-acceptor diblock copolymer

In an argon filled glovebox a solution of **G2** catalyst in anhydrous, degassed THF ([M] = 100 mM) was added into a vial containing cyclophanediene monomer **M1**. The vial was sealed, wrapped in aluminium foil and mixed at room temperature for 5 minutes. The reaction was placed in a preheated drysyn aluminium block at 60 ˚C and stirred for 6 h until consumption of the monomer was observed (SEC and TLC). The reaction mixture was cooled to room temperature and added a solution of the second monomer **M2** in THF (0.3 mL). The reaction mixture was stirred at 60 ˚C for 12 h until the consumption of the monomer **M2** (SEC and TLC) and excess of deoxygenated ethyl vinyl ether was added followed by stirring at room temperature for 6 hours. The crude was precipitated into a short methanol/Celite column, washed with methanol and the polymer was extracted with chloroform. The chloroform layer was evaporated under reduced pressure and dried to obtain desired diblock copolymer (54 mg, 89% yield) as a brown amorphous film.

S8. *Cis-trans* isomerisation

Polymers were dissolved in degassed dichloromethane (1 mg/mL) in an argon filled glovebox. The vial was sealed, removed from the glovebox and subjected to photoisomerisation by irradiating with λ = 365 nm for 48 hours. After evaporation of the solvent polymer were isolated as yellow solids for **8a-c** and **8d** and brown solid for **9** in quantitative yields. ^{1}H NMR spectra of the solid dissolved in CD_2Cl_2 showed complete isomerization of the phenylenevinylene units to *trans* geometry.

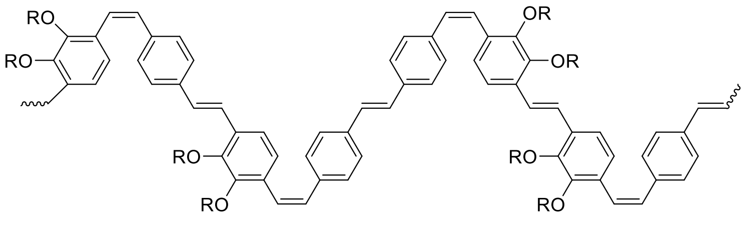

8a-d (n=10-30, R=2-ethylhexyl)

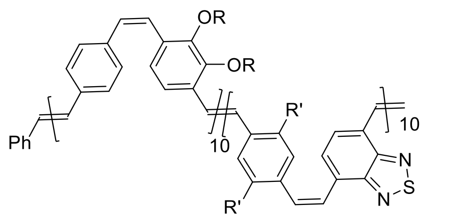

9 (R=2-ethylhexyl, R'= n-octyl)

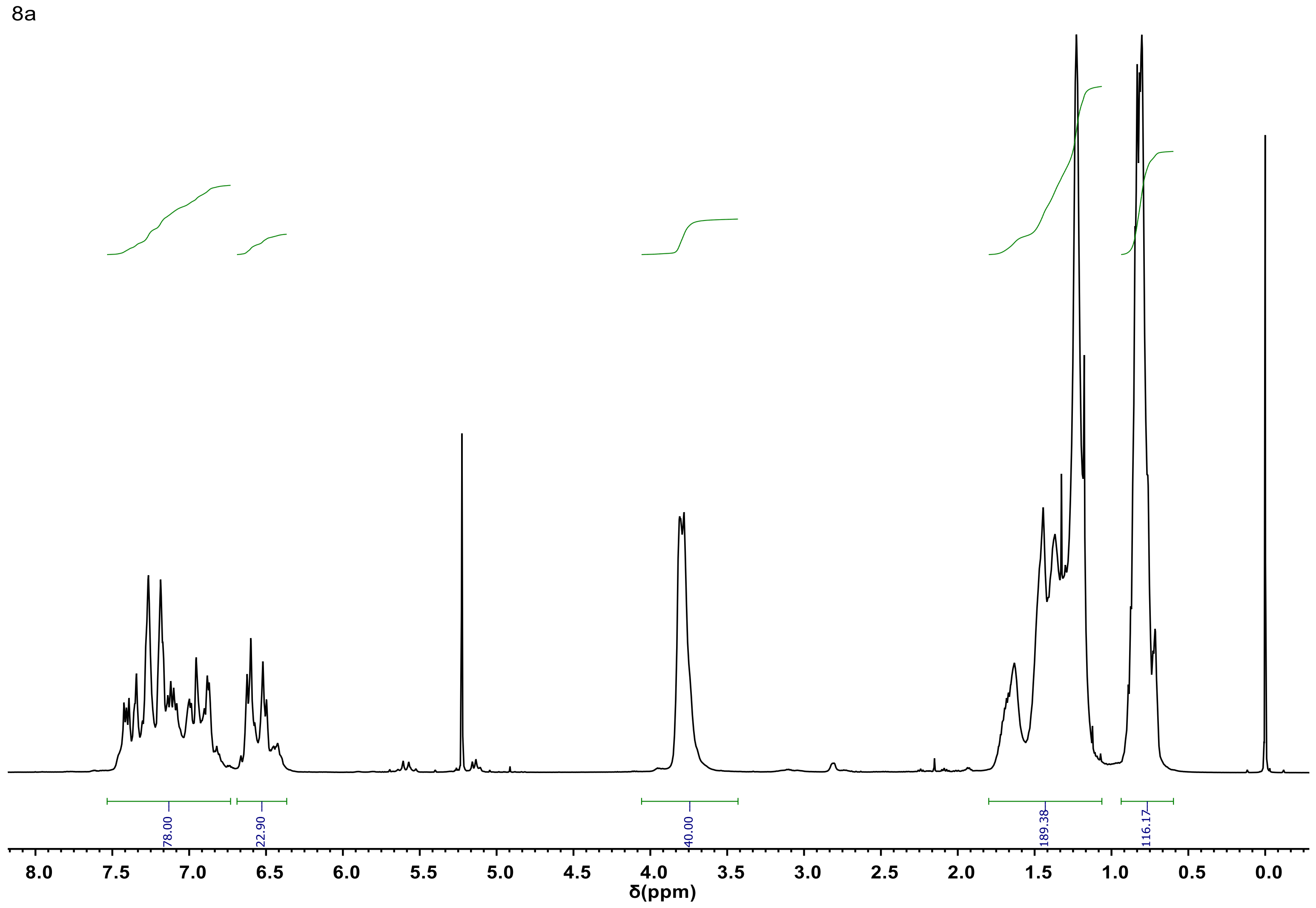

Figure S12 ^{1}H NMR spectrum of **8a** in DCM-d_2.

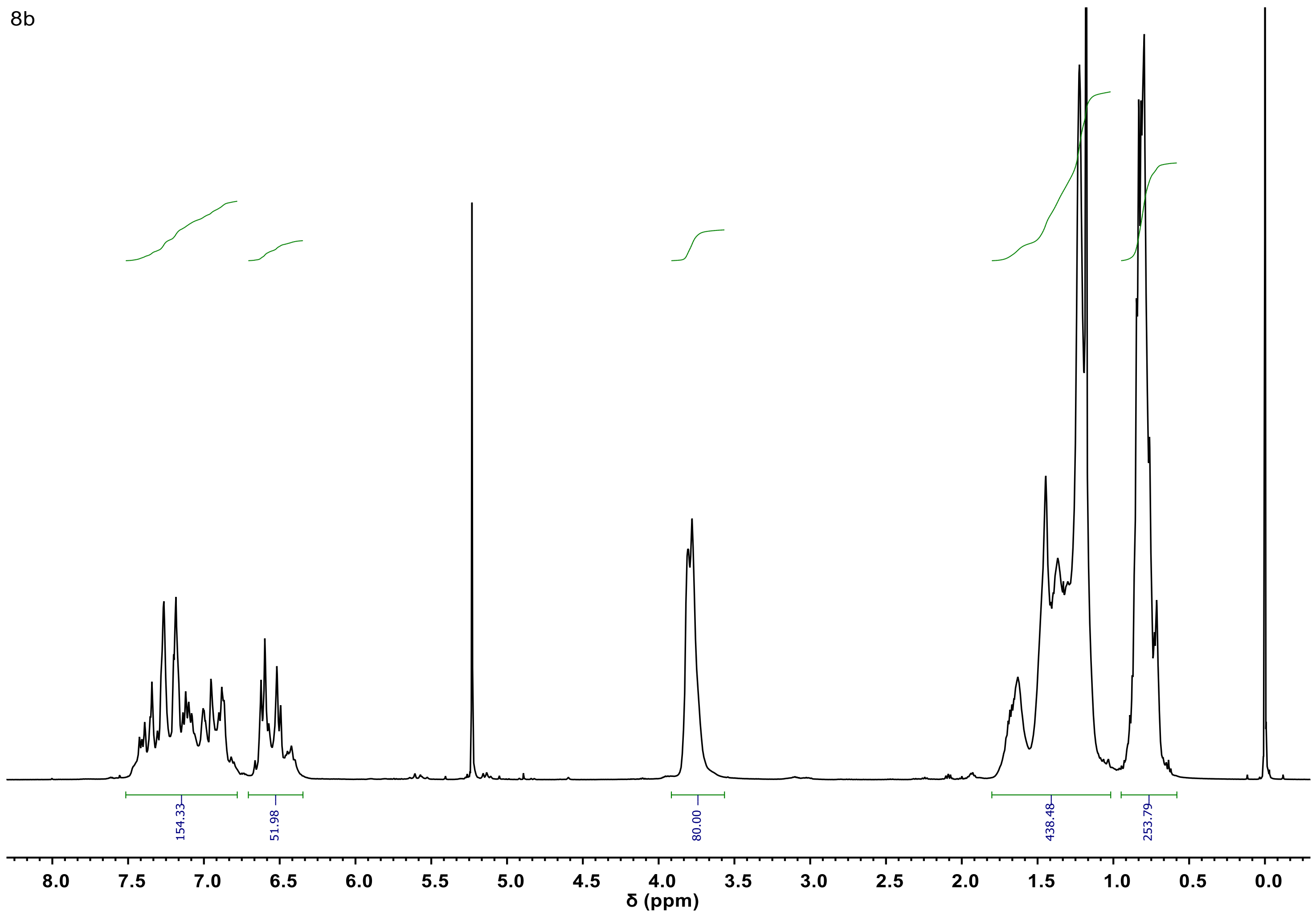

Figure S13 ^{1}H NMR spectrum of **8b** in DCM-d_2.

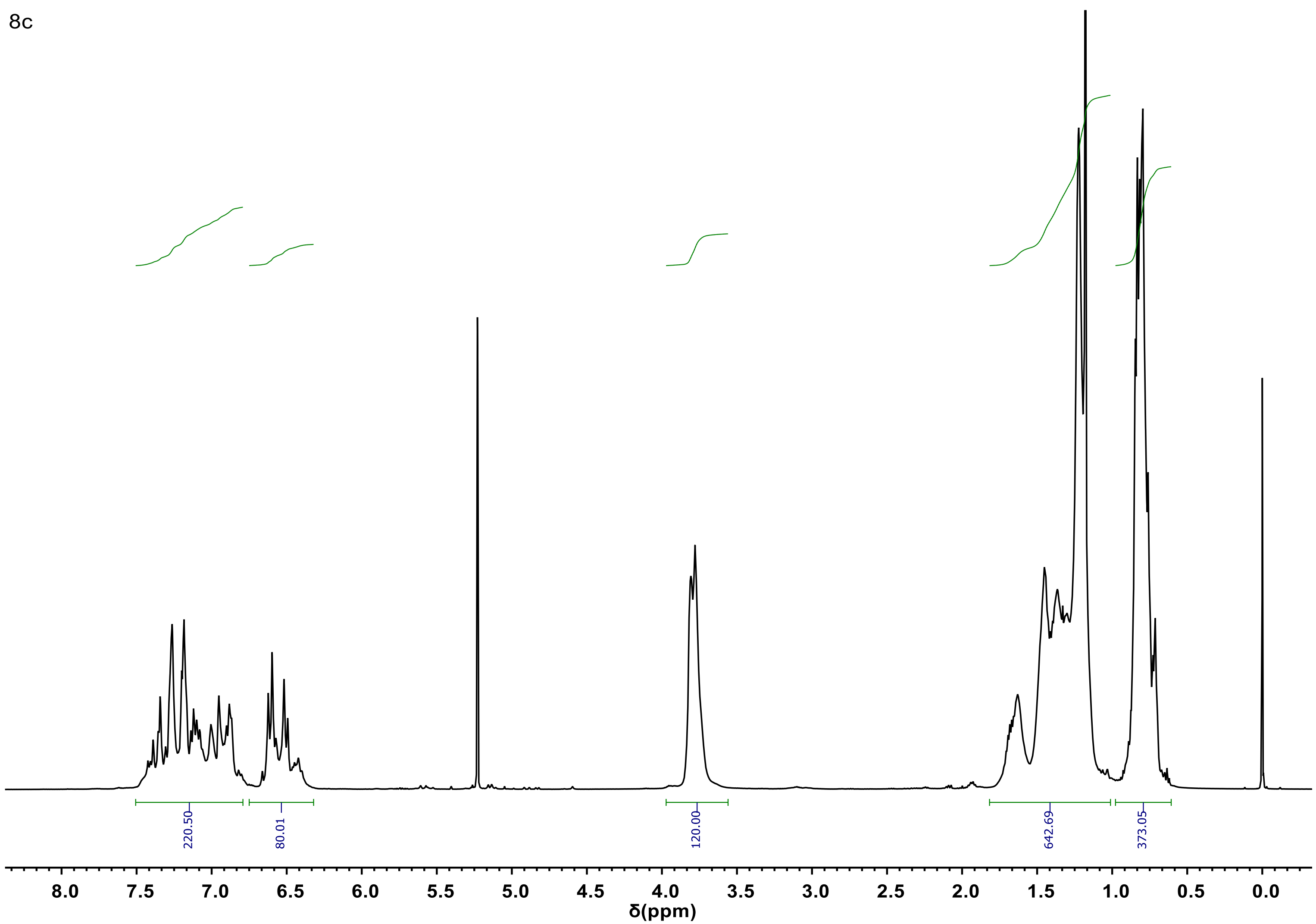

Figure S14 ^{1}H NMR spectrum of **8c** in DCM-d_2.

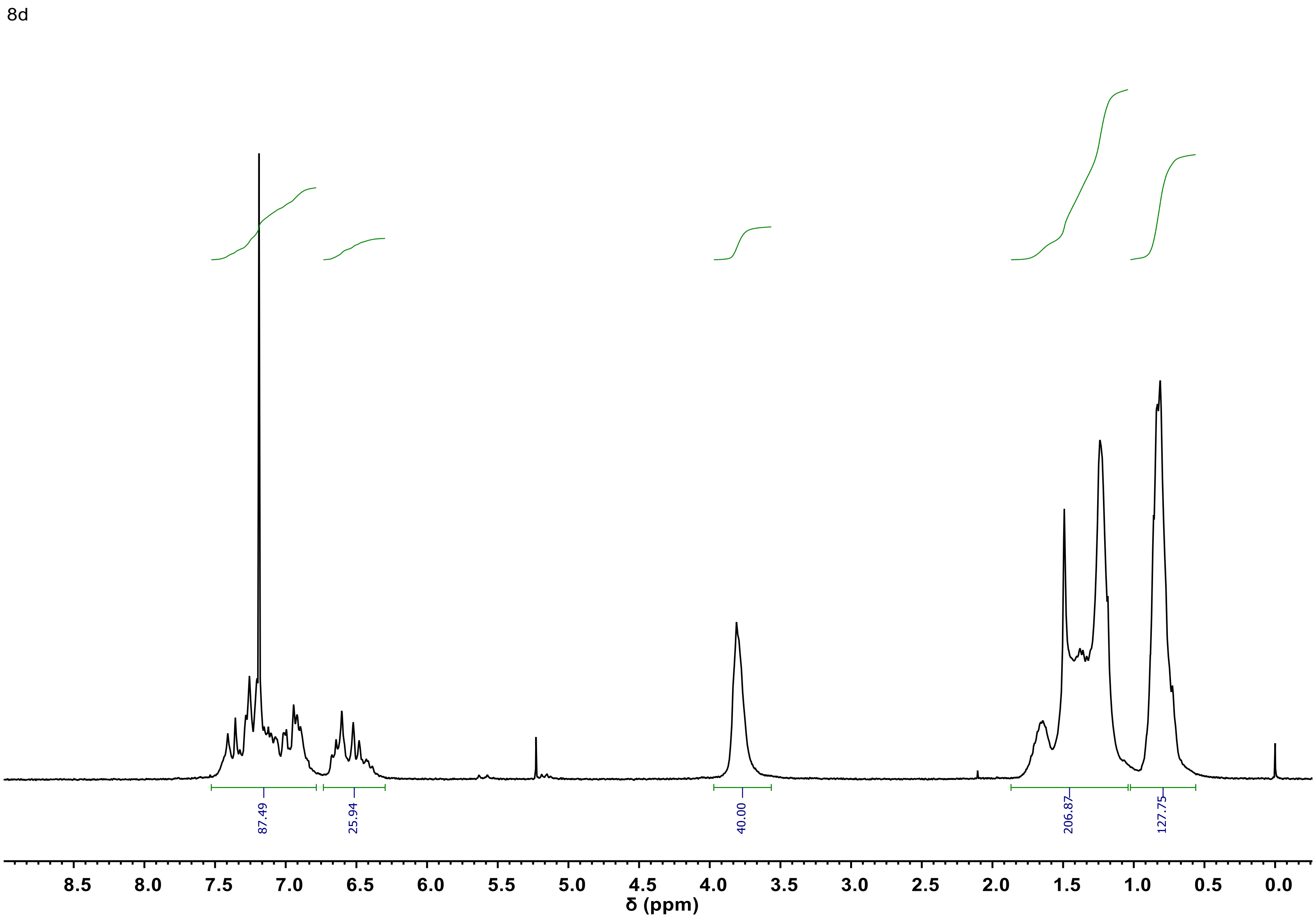

Figure S15 ^{1}H NMR spectrum of **8d** in $CDCl_3$.

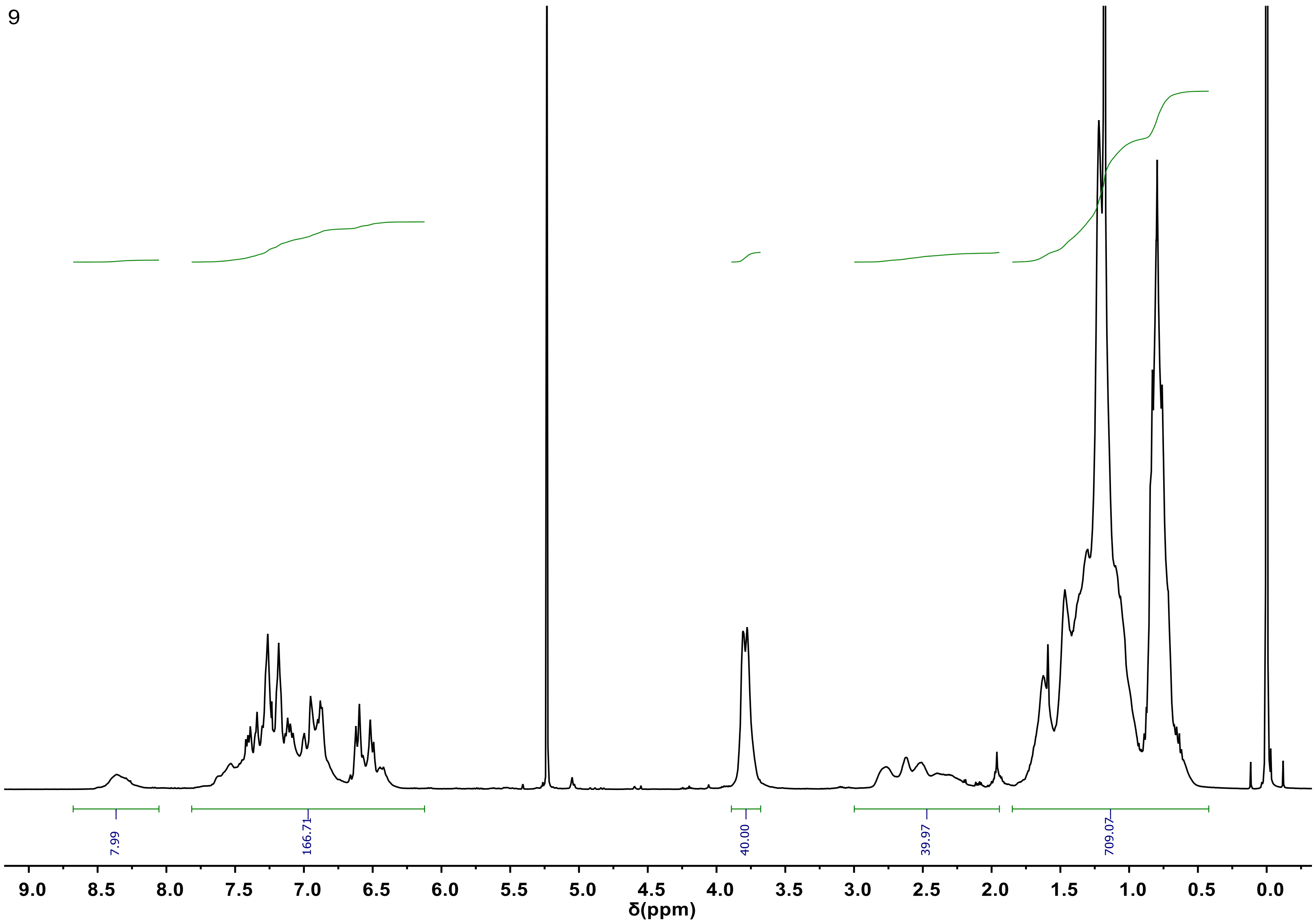

Figure S16 ^{1}H NMR spectrum of **9** in DCM-d_2.

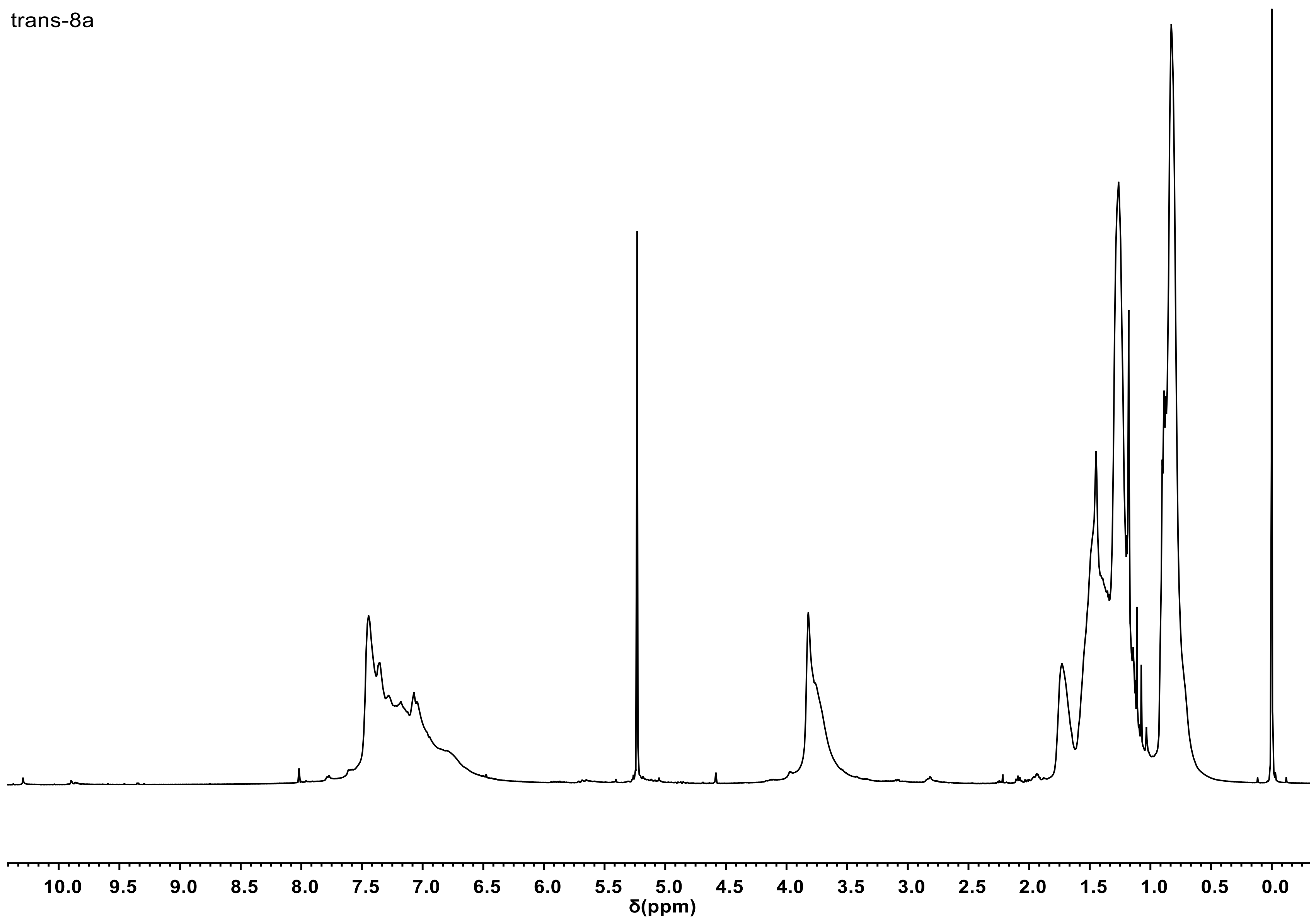

Figure S17 ^{1}H NMR spectrum of *trans*-**8a** in DCM-d_2.

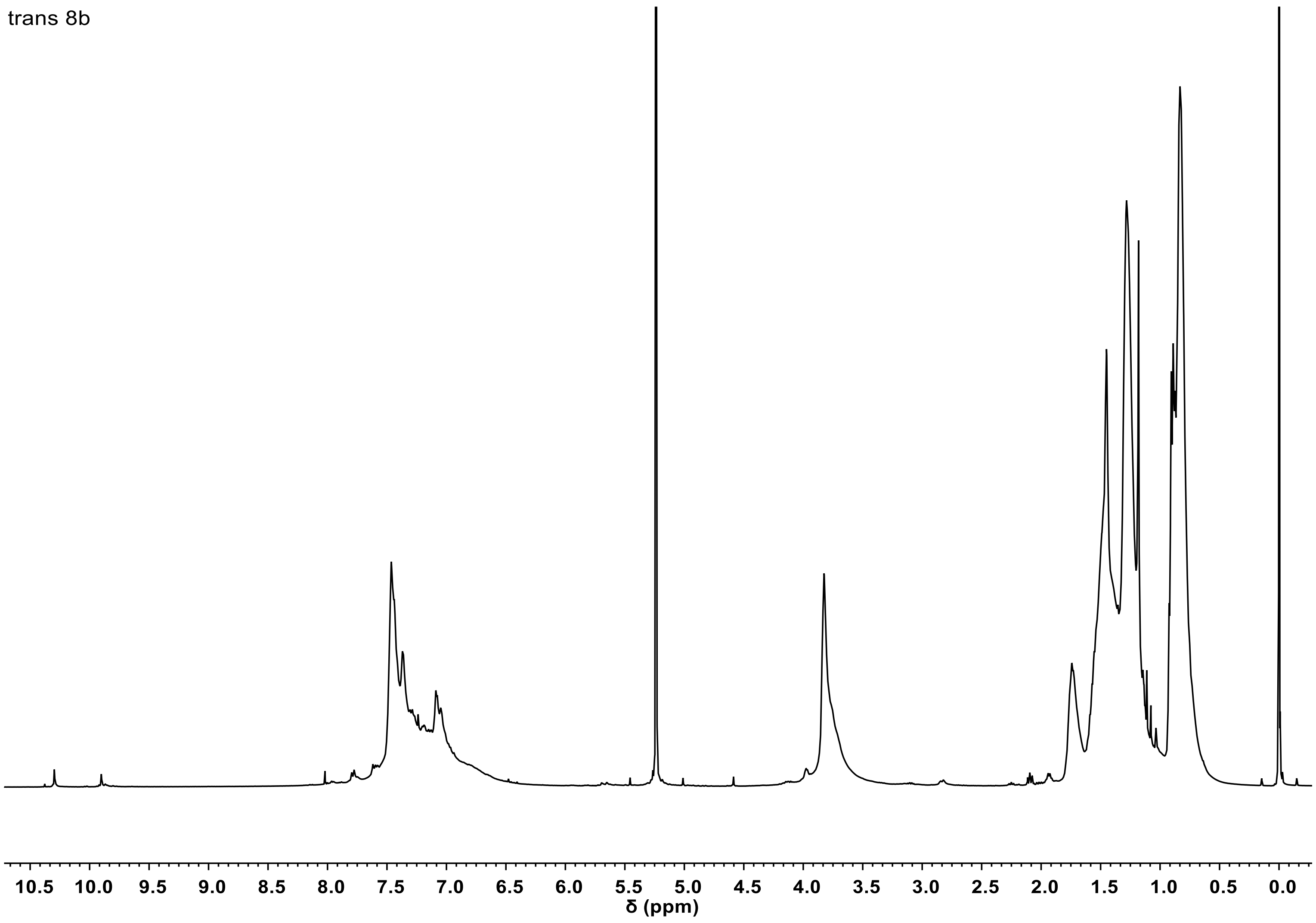

Figure S18 ^{1}H NMR spectrum of *trans*-**8b** in DCM-d_2.

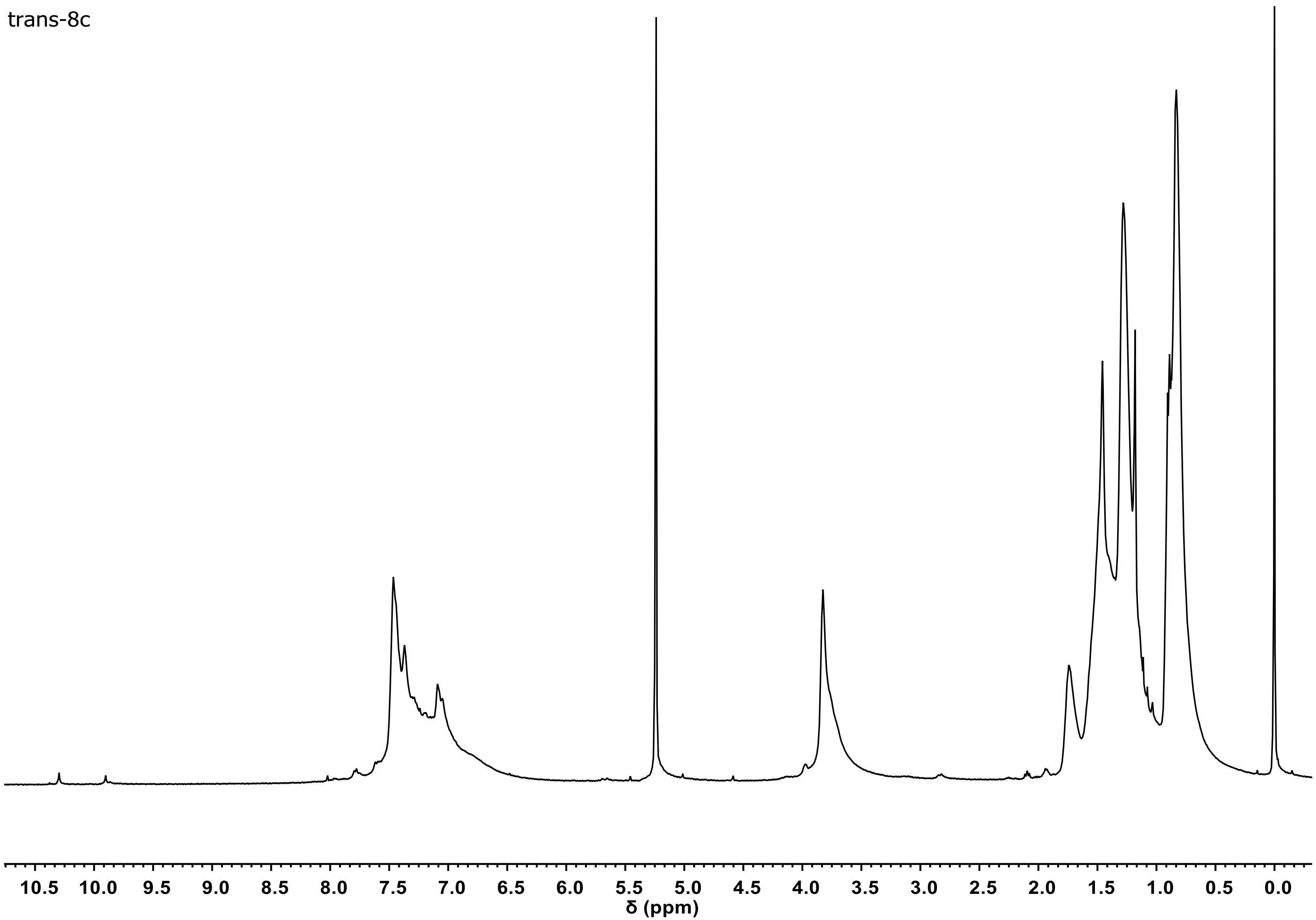

Figure S19 ^{1}H NMR spectrum of *trans*-**8c** in DCM-d_2.

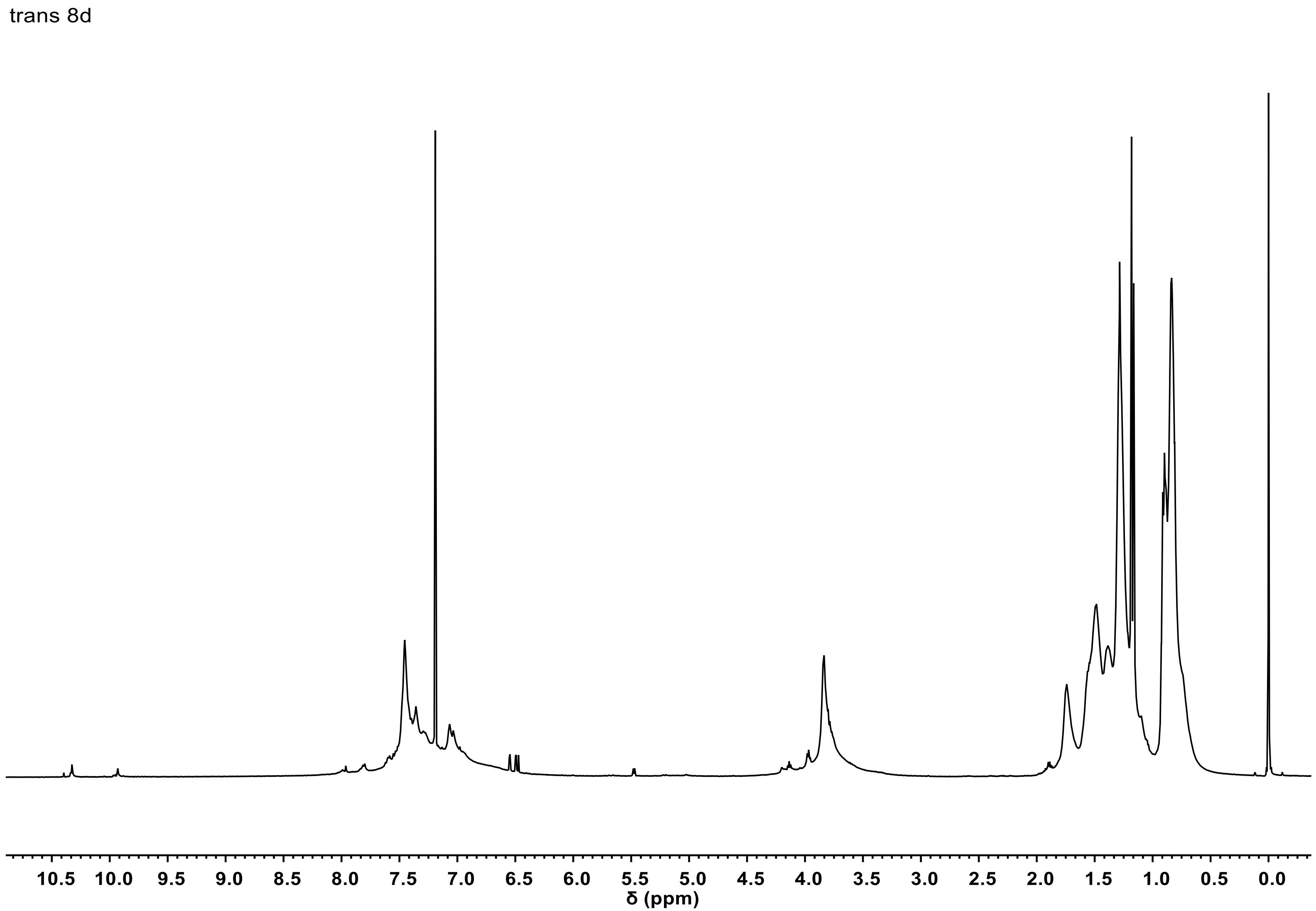

Figure S20 ^{1}H NMR spectrum of *trans*-**8d** in $CDCl_3$.

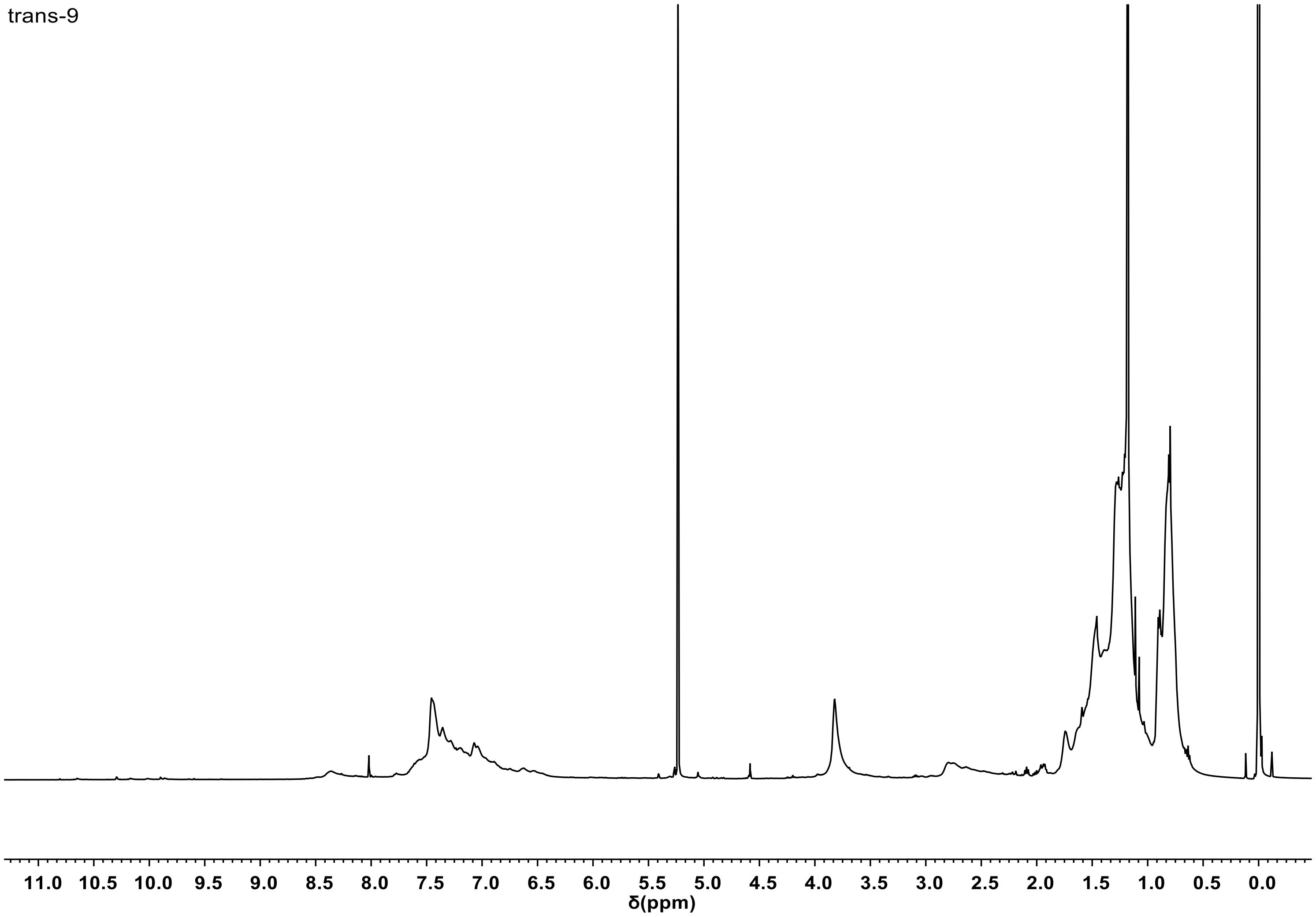

Figure S21 ^{1}H NMR spectrum of *trans*-**9** in DCM-d_2.

S9. Matrix-assisted laser desorption/ionisation time-of-flight mass spectrometry (MALDI-TOF-MS) of polymer 8a

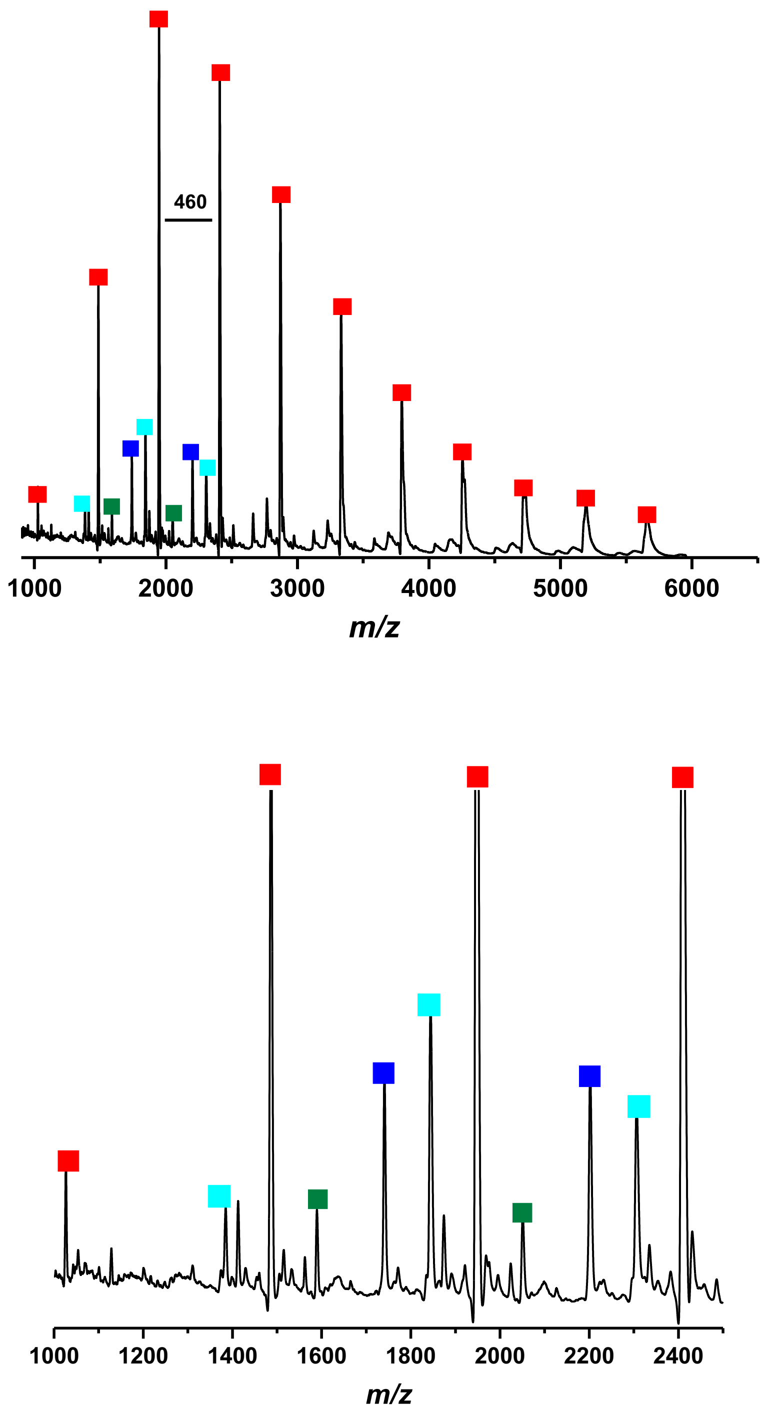

Figure S22 MALDI-TOF mass spectrum of polymer 8a 1000-6000 mass range (top) and 1000-2500 mass range (bottom)

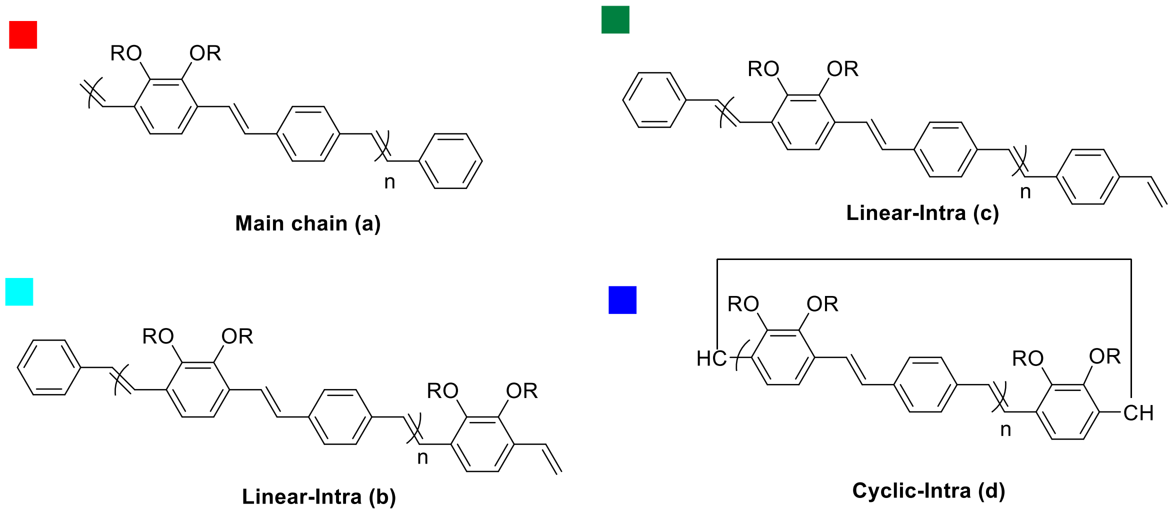

Figure S23 Species produced from *intra* chain metathesis for polymer **8a**

S10. Molecular weight data and SEC traces

Table S1 Molecular weight data for **8a-d** and **9**

	M_n^a	M_n^b	**PDI**	M_n^c
**8a**	4.7	4.9	1.27	4.72
**8b**	9.3	9.4	1.23	9.04
**8c**	14.1	14.9	1.21	15.01
**8d**	4.7	4.7	1.40	6.29
**9**	9.5	11.1	1.40	9.7

[a]Expected M_n (including end groups) [b]Determined by RI detector [c]Determined by ^{1}H NMR

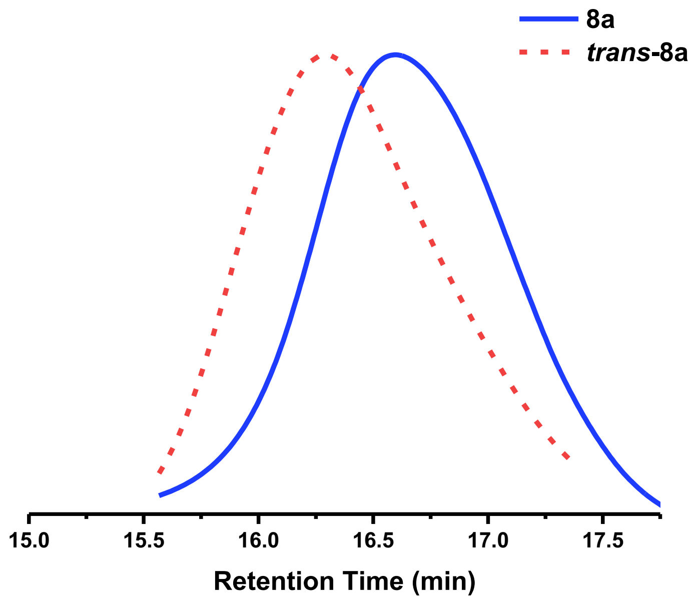

Figure S24 SEC traces of 8a and *trans*-**8a** in THF.

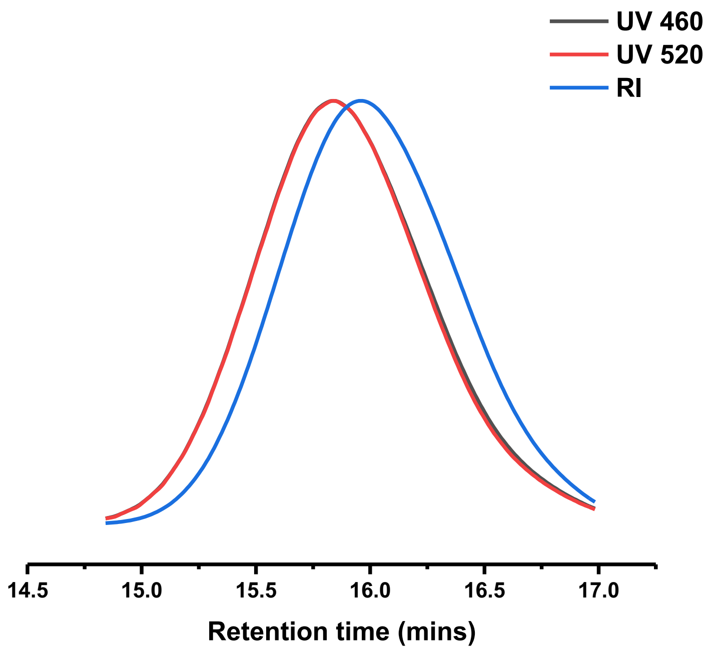

Figure S25 SEC traces (UV and RI) of *cis/trans* BCP **9** in THF.

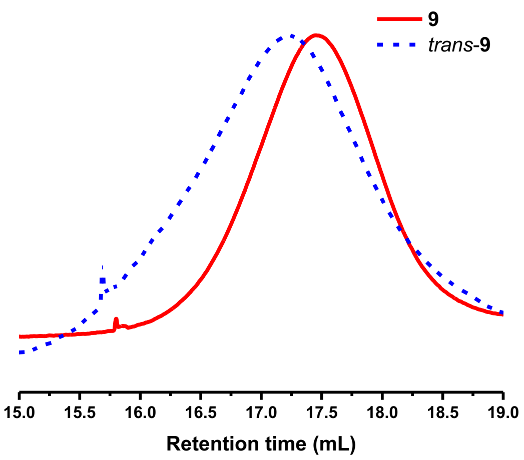

Figure S26 SEC traces of **9** and *trans*-**9** in $CHCl_3$.

S11. Optical properties of polymers

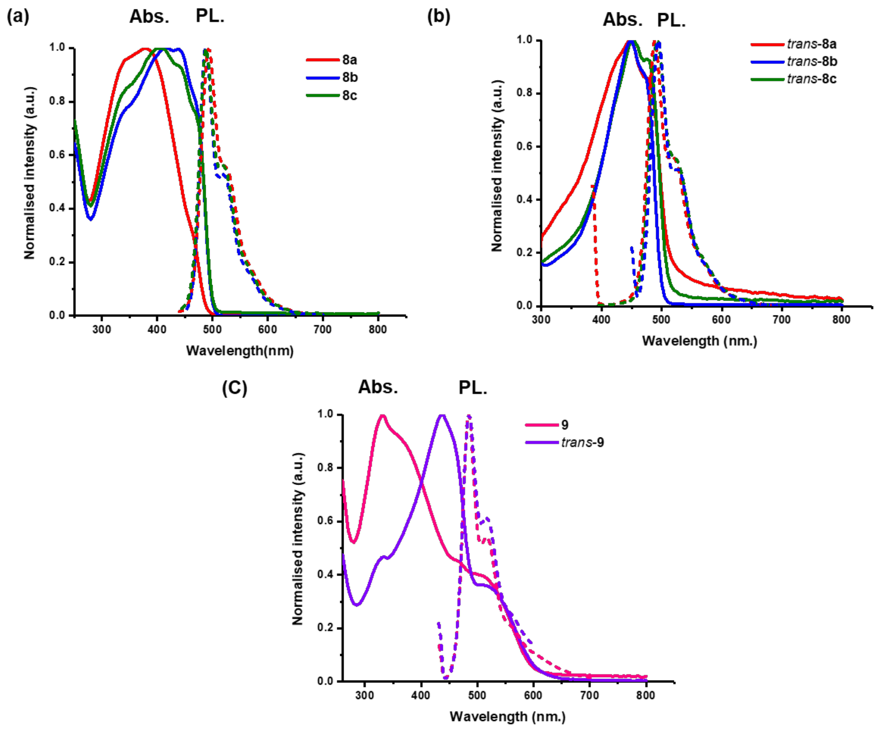

Figure S27 Absorption and emission profiles of copolymers (a) **8a-c** (Ex = 380 nm) , (b) *trans* **8a-c** (Ex = 430 nm) (c) block copolymer **9** (Ex = 330 nm) and *trans*-**9** (Ex = 430 nm) in $CHCl_3$.

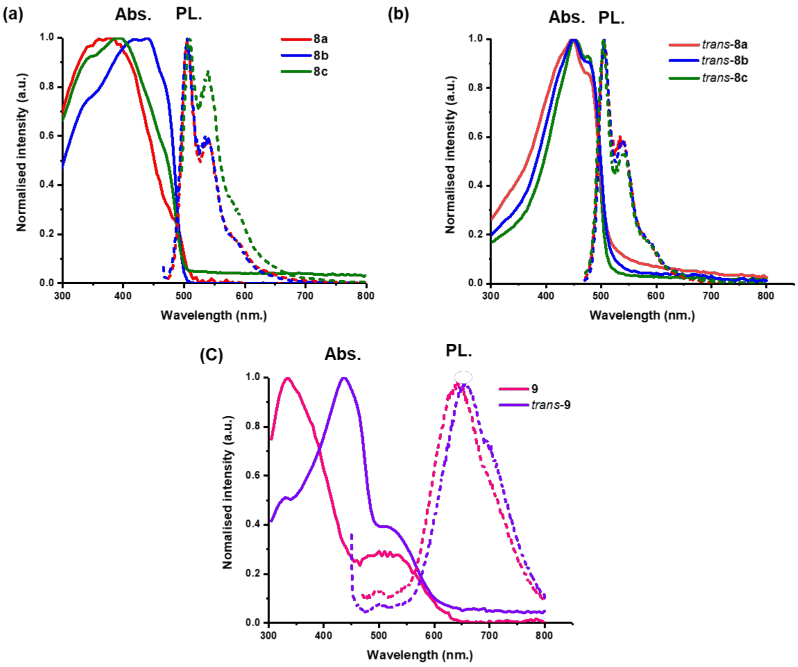

Figure S28 Absorption and emission profiles of copolymers (a) **8a-c** (Ex = 380 nm), (b) *trans* **8a-c** (Ex = 430 nm) (c) block copolymer **9** (Ex = 490 nm) and *trans*-**9** (Ex = 520 nm) in thin film.

Table S2: UV-vis and PL data of polymers in chloroform solution and in solid state

Polymers	$\lambda_{max}(abs.)$ (solution)	$\lambda_{max}(abs.)$ (thin film)	$\lambda(excit.)$	$\lambda_{max}(PL)$ (solution)	$\lambda(excit.)$	$\lambda_{max}(PL)$ (thin film)	$Ø_{PL}$(%)	E_g (eV)[a]
**8a**	384	386	380	490 (546)	380	504(539)	0.64	2.50
***trans*-8a**	438	446	430	498(548)	430	506(540)	0.18	2.43
**8b**	410	414	380	488 (530)	380	505(536)	0.53	2.49
***trans*-8b**	440	447	430	499(549)	430	505(540)	0.19	2.42
**8c**	393	398	380	488 (542)	380	505(539)	0.70	2.49
***trans*-8c**	442	447	430	499(549)	430	508(540)	0.19	2.42
**8d**	376	383(475)	380	484	380	504(539)	0.63	2.51
***trans*-8d**	450(474)	450(481)	440	508(541)	440	528(590)	0.17	2.45
**9**	340, 505	334, 508	330	484 (518)	490	642	0.14	2.20
***trans*-9**	436, 532	438, 540	430	485 (518)	520	658	0.19	2.01

a. E_g=1240/ λ_{onset}

S12. Electrochemical properties of polymers

Table S3 Electrochemical properties of polymers in solid state

**Polymers**	**$[O]_{onset}$ (V)**	**$[R]_{onset}$ (V)**	**HOMO (eV)[a]**	**LUMO (eV)[a]**	**E_g (eV)[b]**
**8a**	1.09	-1.40	5.65	3.16	2.49
**8b**	1.14	-1.32	5.70	3.24	2.46
**8c**	1.09	-1.41	5.65	3.15	2.50
***Trans*-8a**	0.92	-1.30	5.48	3.26	2.22
**9**	0.88	-1.21	5.44	3.35	2.09
***Trans*-9**	0.64	-1.18	5.20	3.38	1.82

a. HOMO= $(E_{ox}^{onset} - Fc_{ox})+4.8$, LUMO= $(E_{red}^{onset} - Fc_{ox})+4.8$, b. $E_{g\,(elc.)}$= HOMO-LUMO

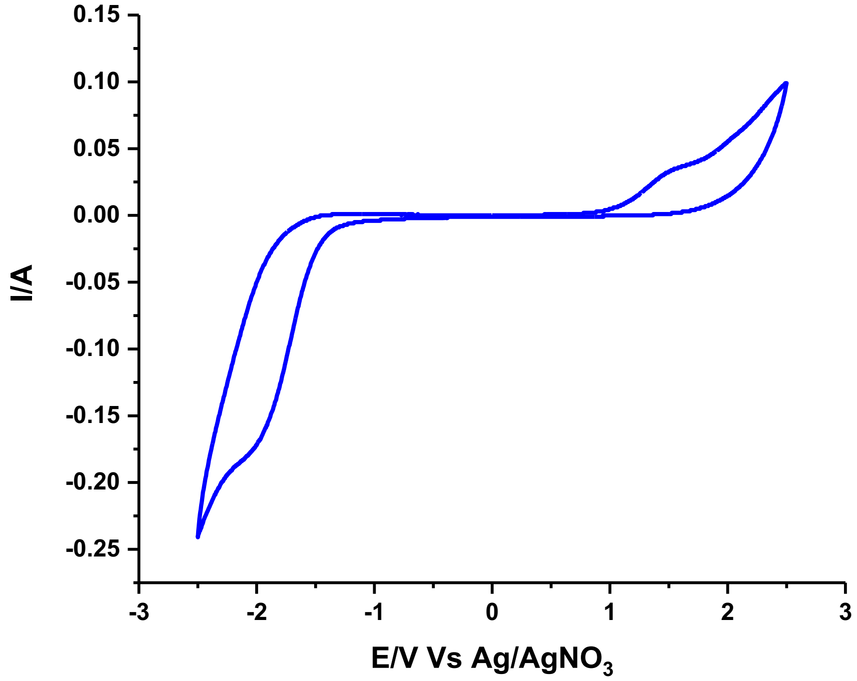

Figure S29 Cyclic voltammograms for **8a** on a Pt electrode in an acetonitrile solution containing 0.1 M *n*-Bu_4NPF.

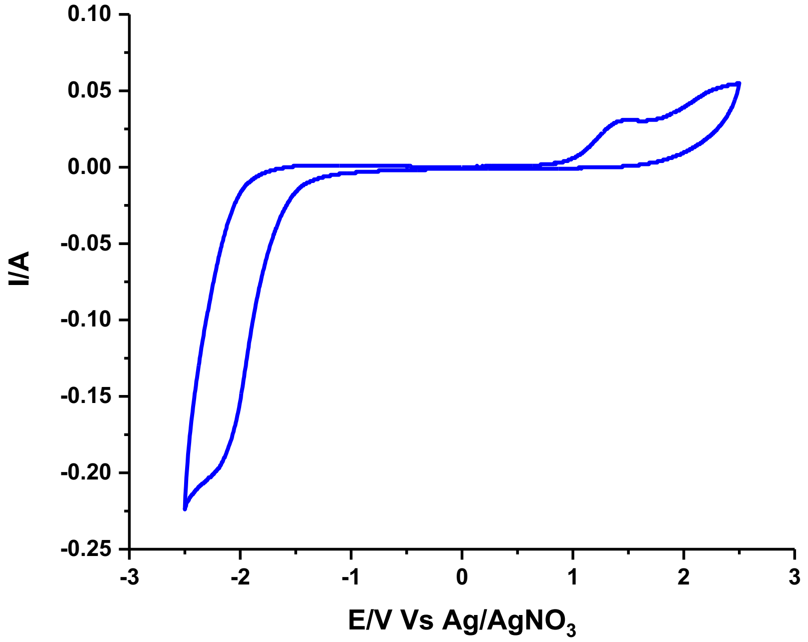

Figure S30 Cyclic voltammograms for **8b** on a Pt electrode in an acetonitrile solution containing 0.1 M *n*-Bu_4NPF.

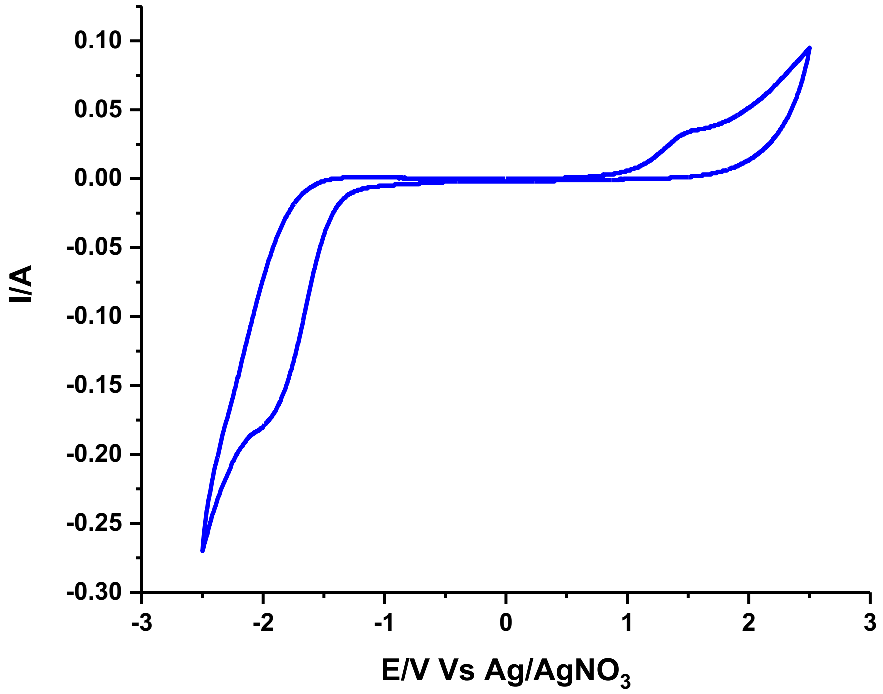

Figure S31 Cyclic voltammograms for **8c** on a Pt electrode in an acetonitrile solution containing 0.1 M *n*-Bu_4NPF.

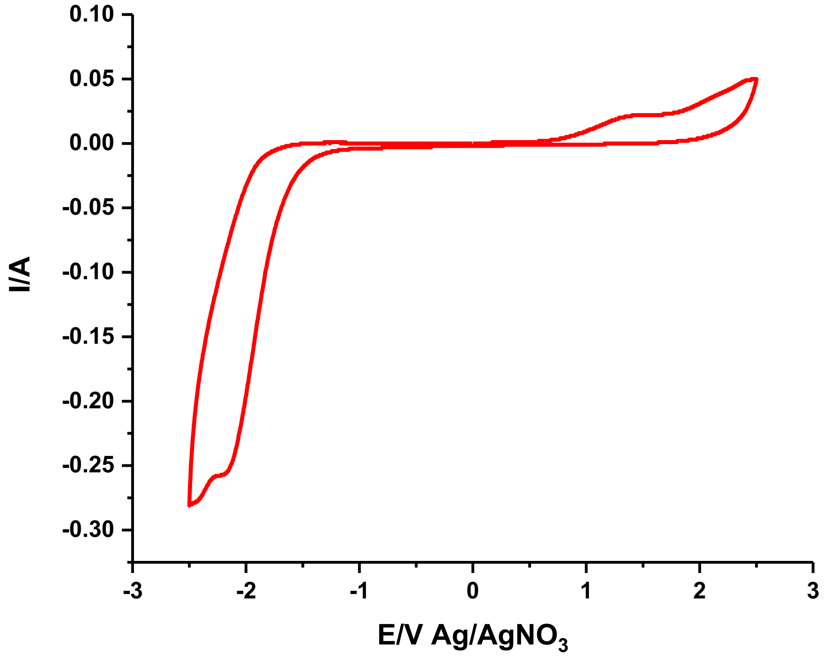

Figure S32 Cyclic voltammograms for *trans*-**8a** on a Pt electrode in an acetonitrile solution containing 0.1 M *n*-Bu4NPF.

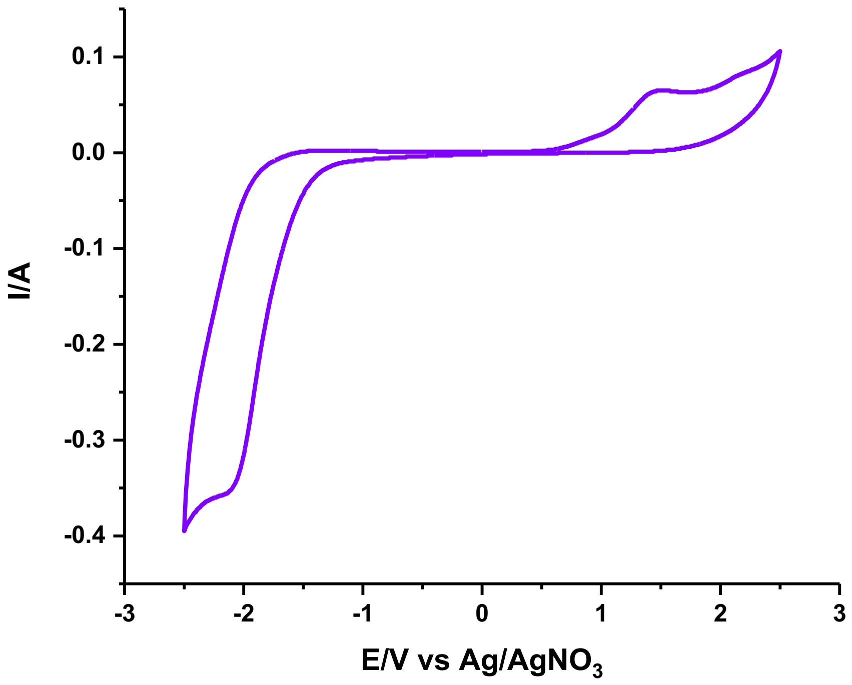

Figure S33 Cyclic voltammograms for **9** on a Pt electrode in an acetonitrile solution containing 0.1 M *n*-Bu4NPF.

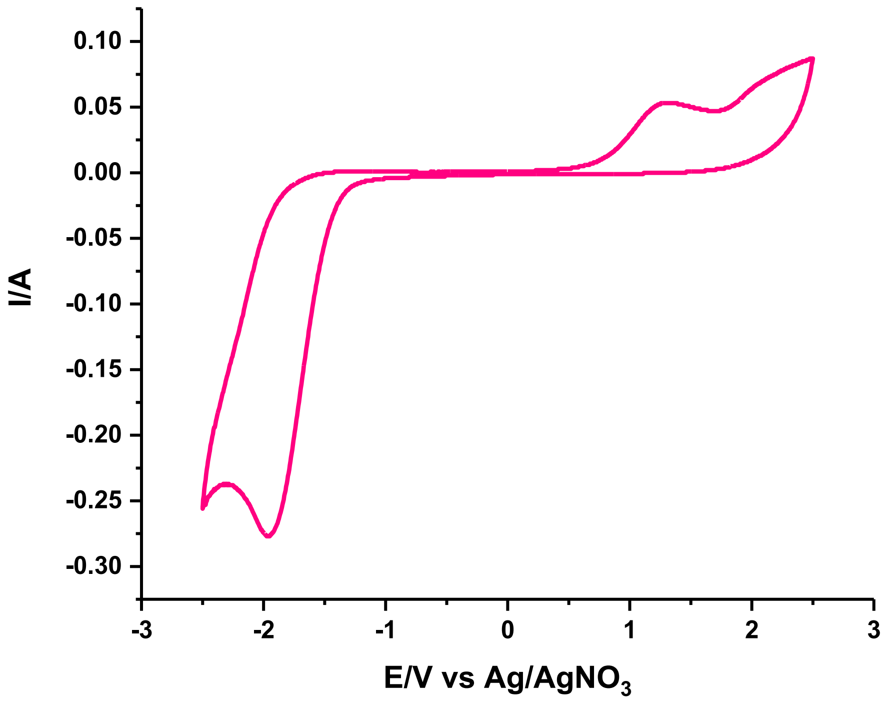

Figure S34 Cyclic voltammograms for *trans*-**9** on a Pt electrode in an acetonitrile solution containing 0.1 M *n*-Bu_4NPF.

S13. References

(1) Kohn, W.; Sham, L. J. Self-Consistent Equations Including Exchange and Correlation Effects. *Phys. Rev.* **1965**, *140* (4A), A1133–A1138. https://doi.org/10.1103/PhysRev.140.A1133.

(2) Lee, C.; Yang, W.; Parr, R. G. Development of the Colle-Salvetti Correlation-Energy Formula into a Functional of the Electron Density. *Phys. Rev. B* **1988**, *37* (2), 785–789. https://doi.org/10.1103/PhysRevB.37.785.
